# On Differentiating Multiple Types of ULF Magnetospheric Waves in Response to Solar Wind Periodic Density Structures

**DOI:** 10.1029/2021JA030144

**Published:** 2022-03-22

**Authors:** S. Di Matteo, U. Villante, N. Viall, L. Kepko, S. Wallace

**Affiliations:** ^1^ Department of Physics The Catholic University of America Washington DC USA; ^2^ NASA Goddard Space Flight Center Greenbelt MD USA; ^3^ Department of Physical and Chemical Sciences University of L’Aquila L’Aquila Italy; ^4^ Consorzio Area di Ricerca in Astrogeofisica L’Aquila Italy

**Keywords:** periodic density structures, ULF waves at discrete frequencies, radiation belts, solar wind discontinuities, solar wind formation, multitaper spectral analysis

## Abstract

Identifying the nature and source of ultra‐low frequencies (ULF) waves (*f* ⪅ 4 mHz) at discrete frequencies in the Earth's magnetosphere is a complex task. The challenge comes from the simultaneous occurrence of externally and internally generated waves, and the ability to robustly identify such perturbations. Using a recently developed robust spectral analysis procedure, we study an interval that exhibited in magnetic field measurements at geosynchronous orbit and in‐ground magnetic observatories both internally supported and externally generated ULF waves. The event occurred on 9 November 2002 during the interaction of the magnetosphere with two interplanetary shocks that were followed by a train of 90 min solar wind periodic density structures. Using the Wang‐Sheeley‐Arge model, we mapped the source of this solar wind stream to an active region and a mid‐latitude coronal hole just prior to crossing the Heliospheric current sheet. In both the solar wind density and magnetospheric field fluctuations, we separated broad power increases from enhancements at specific frequencies. For the waves at discrete frequencies, we used the combination of satellite and ground magnetometer observations to identify differences in frequency, polarization, and observed magnetospheric locations. The magnetospheric response was characterized by: (a) forced breathing by periodic solar wind dynamic pressure variations below ≈1 mHz, (b) a combination of directly driven oscillations and wave modes triggered by additional mechanisms (e.g., shock and interplanetary magnetic field discontinuity impact, and substorm activity) between ≈1 and 4 mHz, and (c) largely triggered modes above ≈4 mHz.

## Key Points

- First robust identification of the periodic density structures’ solar source region for an event in which they drove magnetospheric dynamics
- Periodic density structures impact resulted in magnetosphere dynamics including directly driven ultra‐low frequency (ULF) waves, field line resonance, and local changes in radiation belt particle flux
- Interplanetary magnetic field discontinuities at the border of density structures might also trigger Pc5 ULF waves

## 1 Introduction

Ultra low frequencies (ULF) waves in the Earth's magnetosphere are magnetic field fluctuations ranging from a few mHz to Hz. They were first classified in terms of frequency and whether the waveforms were continuous (Pc) or irregular (Pi; Jacobs et al., 1964). Pc5 ULF waves are a subset comprising continuous pulsations with frequencies in the ≈1.7–6.7 mHz band. Many generation mechanisms have been proposed to explain their characteristics, including: Kelvin‐Helmholtz instability at the magnetopause flanks (Chen & Hasegawa, 1974; Southwood, 1974); impact onto the magnetosphere of interplanetary shocks or pressure impulses (Allan et al., 1986; Mann et al., 1998; Southwood & Kivelson, 1990); solar wind buffeting (Wright & Rickard, 1995); surface modes at the magnetopause (Archer et al., 2013, 2019; Archer & Plaschke, 2015; Plaschke & Glassmeier, 2011) and the plasmapause (He et al., 2020; Nenovski, 2021); transient ion foreshock phenomenon (Hartinger et al., 2013; B. Wang et al., 2020); and resonance with injected energetic particles (Glassmeier et al., 1999; James et al., 2013; Yeoman et al., 2010). Some of these processes involve the coupling of fast magnetosonic waves with shear Alfvén waves in the field line resonance (FLR) process (Chen & Hasegawa, 1974; Southwood, 1974) and/or cavity/waveguide modes (Harrold & Samson, 1992; Hartinger et al., 2012; Kivelson & Southwood, 1985, 1986; Mann et al., 1999; Rickard & Wright, 1994; Samson et al., 1992; Wright, 1994). The Pc5 waves can also result from direct driving of the magnetospheric fluctuations by solar wind periodic density structures (PDS). The PDSs manifest in the solar wind as density fluctuations at frequencies typically below 4.0 mHz. At nominal solar wind speeds, the PDSs correspond to structures with size scales of the order of the Earth's magnetosphere (Kepko et al., 2020). The resultant directly driven ULF waves, observed in the magnetosphere and at the ground, occur at similar frequencies falling within and extending beyond the Pc5 band (Birch & Hargreaves, 2020; Hartinger et al., 2014; Kepko et al., 2002; Kepko & Spence, 2003; Stephenson & Walker, 2002; Viall et al., 2009; Villante et al., 2016).

Structures in the solar wind can be either injected remnants of solar corona processes or locally generated in situ by dynamical process en route to the observation point (Borovsky, 2021; Viall et al., 2021). Many statistical and case studies have shown that the solar wind at 1 AU contains periodic proton density structures at length scales that occur more often than others (Kepko et al., 2020, and references therein). In the rest frame of a spacecraft or Earth, the structure's length scale (*L*) and the solar wind velocity (*v*) determine the apparent frequency of the density fluctuations (*f* = *v*/*L*). These periodic density structures have been observed both in remote and in situ data. Viall and Vourlidas (2015) found that PDSs are created at the Sun as the solar wind is formed and exhibit a typical periodicity of ≈90 min (Viall et al., 2010). Their signatures have been observed at 0.3, 0.4, and 0.6 AU using in situ data from Helios 1 and Helios 2 (Di Matteo et al., 2019) as well as at 1 AU (Kepko et al., 2016) and beyond (Birch & Hargreaves, 2021). These events are consistent with recent simulations showing that the tearing instability and magnetic reconnection at the tip of the helmet streamer can release coronal plasma in “bunches” with a typical periodicity of ≈80 min (Réville et al., 2020). Nevertheless, the PDSs are not limited to the heliospheric current sheet (HCS), but they constitute a fair portion of the fast solar wind and can occur in up to 80% of the slow solar wind at 1 AU (Kepko et al., 2020; Viall et al., 2008).

Pc5 waves can also manifest at sets of discrete frequencies. Originally, Samson et al. (1991); Samson et al. (1992), Ruohoniemi et al. (1991), and Walker et al. (1992), identified in the northern auroral region oscillations at *f* ≈ 1.3, ≈1.9, ≈2.6–2.7, and ≈3.2–3.4 mHz in the F region drift velocities (Goose Bay Radar) and in the geomagnetic field components from the Canadian Auroral Network for the OPEN Program Unified Study (CANOPUS) magnetometer array. These modes were interpreted in terms of FLRs driven by waveguide/cavity modes of the magnetosphere, possibly excited by solar wind dynamic pressure pulses or the Kelvin‐Helmholtz instability at the magnetopause. However, statistical surveys at the same site found that the proposed set of discrete frequencies was not particularly distinguished from other repeated frequencies (G. J. Baker et al., 2003; Ziesolleck & McDiarmid, 1995). Nevertheless, analysis at other sites reported similar sets of repeated frequencies (Chisham & Orr, 1997; Francia et al., 2005; Francia & Villante, 1997; Mathie et al., 1999; Norouzi‐Sedeh et al., 2015; Provan & Yeoman, 1997; Villante et al., 2001, 2016). One of the major challenges in the study of this phenomena comes from the ability to robustly identify such discrete oscillations, since the use of different analysis techniques and selection criteria can lead to the identification of different sets of discrete frequencies (Di Matteo & Villante, 2017, 2018).

Recently, surface wave modes have been linked to ULF waves at discrete frequencies at the magnetopause and the plasmapause. He et al. (2020) showed that MHD surface waves at ≈ 1.4 and ≈2.2 mHz supported by the plasmapause are observed at ground observatories. Archer et al. (2019) identified signatures of magnetopause surface modes at ≈1.7–1.8 and ≈3.3 mHz in satellite observations accompanied by some evidence of fluctuations at ≈ 3.5 ± 0.2 mHz in ground magnetometer. Note that magnetopause surface eigenmodes can also drive waves at a frequency below the Pc5 band (Archer et al., 2013; Plaschke et al., 2009). Although this observational evidence supports the surface wave mode hypothesis, their signatures at ground observatories is still unclear. Long lasting Pc5 waves at high latitude are unlikely to be signatures of surface mode waves (Pilipenko et al., 2017, 2018), while short lived ones have similar signatures to heavily damped Alfvénic oscillations of the last closed field lines (Kozyreva et al., 2019). While MHD surface modes on one plasma boundary appear to be localized at the ground (He et al., 2020), surface modes common to two plasma boundaries, that is, the magnetopause and the plasmapause, have been suggested as a possible mechanism for global ULF waves at several discrete frequencies below ≈4 mHz (Nenovski et al., 2007). However, the persistence of these surface modes depends on the conditions of the magnetosphere and their source (Nenovski, 2021).

Each source of ULF oscillations has different characteristics, and we lack a conclusive explanation for the simultaneous appearance at high, mid, and low latitudes of ULF waves at several discrete frequencies below ≈4 mHz. One possible reason could be the intrinsic simultaneous occurrence of many generation mechanisms. Previous analyses investigating the role of PDSs in the generation of ULF waves were focused on the one‐to‐one correspondence between solar wind density and global magnetospheric field fluctuations at specific frequencies. However, while magnetospheric field fluctuations at the longer time scales (i.e., with frequencies below ≈1 mHz) can be treated as quasi‐static modulation of the magnetosphere by the slowly varying solar wind dynamic pressure, oscillations between ≈ 1 mHz and ≈4 mHz are associated with structures of size scales on the same order of the Earth's magnetosphere cavity. Therefore, the chain of interaction between smaller PDSs might involve multiple additional magnetosphere responses. Takahashi and Ukhorskiy (2007) suggested three different solar wind/magnetosphere coupling processes in the generation of ULF waves controlled by dynamic pressure fluctuations: (a) compressing and relaxing the magnetospheric cavity in a “forced breathing” mode; (b) controlling the position of the magnetopause, and thereby the amplitude of waves observed in the magnetosphere created by magnetopause surface waves; (c) buffeting of the magnetosphere that generates fast magnetosonic waves which then couple to toroidal standing Alfvén waves. As a step forward to a better understanding of these processes, we investigate in detail the properties of ULF waves occurring on 9 November 2002, during the interaction of the magnetosphere with a complex interplanetary structure, characterized by two consecutive interplanetary shocks followed by PDSs. First, we analyzed observations of the solar wind and identified the stream source on the Sun. Then, we used observations from geostationary satellites and ground magnetometers to characterize the magnetospheric ULF wave activity and some of the resultant effects on the radiation belt electrons.

## 2 Data and Methods

We used solar wind density measurements from the Solar Wind Experiment instrument (SWE; Ogilvie et al., 1995) and interplanetary magnetic field from the Magnetic Field Instrument (MFI; Lepping et al., 1995) onboard the Wind spacecraft. We considered the solar wind proton (*n*_*p*_) and alpha (*n*_*α*_) number density; their ratio (*n*_*α*_/*n*_*p*_), the solar wind speed (*v*) and dynamic pressure (*Dp*); the interplanetary magnetic field (IMF) intensity (*B*) and its direction through *Θ*_*B*_ = arcsin(*B*_*z*_/*B*) and *Φ*_*B*_ = arctan(*B*_*y*_/*B_x_*) in the Geocentric Solar Magnetospheric (GSM) coordinate system; the thermal pressure (*p*_*T*_, including measured *α* and electrons), the magnetic pressure (*p*_*B*_), and the total pressure (*p*_*tot*_); and the plasma beta value (*β* = *p*_*tot*_/*p*_*B*_). We also used the Wang‐Sheeley‐Arge (WSA) model (Arge & Pizzo, 2000; Arge et al., 2003, 2004) to estimate the source region of the solar wind stream. WSA couples two magnetostatic potential‐field type models (Altschuler & Newkirk, 1969) to derive the Sun's coronal magnetic field from 1 to 5 solar radii (*R*_⊙_). The location of Wind is then projected back to the outer coronal boundary of the model at 5 *R*_⊙_, and matched with the corresponding endpoints of the coronal magnetic field lines. The model then propagates individual solar wind parcels from the endpoints of those field lines out to 1 AU to determine the time of arrival of the solar wind at Wind. Thus, the field lines and solar wind stream observed at Wind can be traced back to 1 *R*_⊙_ to reveal the sources of the solar wind. We derived coronal magnetic field solutions for this study using synchronic photospheric field maps generated by the Air Force Data Assimilative Photospheric Flux Transport (ADAPT) model (Arge et al., 2010, 2011, 2013; Hickmann et al., 2015), using observations from the Kitt Peak Vacuum Telescope (KPVT: Jones et al. [1992]). For more details on this methodology, see Wallace et al. (2020).

We investigated the response of the magnetosphere using the averaged one‐minute magnetospheric field observations in the ENP coordinate system at geostationary orbit from the fluxgate magnetometers (MAG; Singer et al. [1996]) onboard the Geostationary Operational Environmental Satellites (GOES), specifically from GOES 8 and GOES 10. The *H*_*p*_ component is perpendicular to the satellite orbit and directed northward; the *H*_*n*_ component is along the satellite trajectory and positive eastward, the *H*_*e*_ component completes the triad and is directed earthward. Given the position of the satellites, the three components can be interpreted respectively as the compressional, toroidal, and poloidal components of the magnetospheric field.

We complemented these observations with magnetic field measurements at all ground magnetic observatories available from the SuperMAG collaboration, listed in the Supporting Information. We used the 60 s resolution vector magnetic field in the NEZ coordinate system where *B*_*N*_ is directed toward magnetic north, *B*_*E*_ toward magnetic east, and *B*_*Z*_ is vertically down. The daily variations and yearly trends determined by the Gjerloev (2012) algorithm were subtracted from each component. To monitor the magnetospheric conditions we collected the sym‐H and AE indices.

We investigated the energetic particle response using measurements from the Los Alamos National Laboratory (LANL) Synchronous Orbit Particle Analyzer (SOPA) detector (Belian et al., 1992) and Energy Spectrometer for Particles (ESP) instrument (Meier et al., 1996) on board LANL–01A (LT = UT+00:31), LANL–02A (LT = UT+04:42), LANL–97A (LT = UT+06:55), 1994–084 (LT = UT+09:43), 1991–080 (LT = UT–11:02), and 1990–095 (LT = UT–02:34) satellites. We considered the one‐minute electron particle flux data, averaged for six ≈10 s data accumulation cycles, for 15 differential electron channels: nine from the SOPA detector (51–77, 77–107, 107–151, 151–226, 226–316, 316–500, 500–750, 750–1090, 1090–1540 keV), and six from the ESP instrument (0.7–1.8, 1.8–2.2, 2.2–2.7, 2.7–3.5, 3.5–4.5, 4.5–6.0 MeV).

In order to identify ULF fluctuations in the time series, we use the spectral analysis procedure described in Di Matteo et al. (2021). Briefly, we used the statistical properties of the adaptive multitaper (MTM; Thomson, 1982) power spectral density (PSD) estimates to perform the maximum likelihood fitting of PSD background models and determine the confidence thresholds for PSD outliers (*γ* test). In this work, we tested power law and bending power law models on the raw and bin‐smoothed PSD (Di Matteo et al., 2021). The results are combined with the harmonic *F* test, an additional statistical test deriving from a complex‐valued regression analysis, searching for F value peaks at frequencies within the PSD enhancements (*γ* + F test). Note that in case of rapid evolution of the periodicity (on timescales smaller than the window in analysis) or the occurrence of multiple signals at frequencies within a power enhancement, the *F* test can identify multiple peaks (Di Matteo & Villante, 2017). For all the observations, we evaluated the dynamic spectrum and *F*‐test values considering linear detrended time series from a ≈91 min sliding window. We applied the MTM with time‐halfbandwidth product *NW* = 3 and number of tapers *K* = 4, selecting peaks in the PSD and the *F* test above the 90% confidence level. While the use of a single selection criterion would result in a false positive rate of 10%, the combined amplitude + *F*‐test provides false positive rates lower than 2%, as demonstrated by Monte Carlo simulation of synthetic time series (Di Matteo et al., 2021). However, due to border effects, this is valid only in a restricted frequency range away from the frequency bounds, namely [2*NWf*_*Ray*_, *f*_*Ny*_ − 2*NWf*_*Ray*_], where Δ*t* is the sampling time, *f*_*Ny*_ = 1/2Δ*t* is the Nyquist frequency, and *f*_*Ray*_ = 1/*N*Δ*t* is the Rayleigh frequency.

We interpolated the Wind observations to the average sampling time for the interval of interest, ≈97 s, corresponding to a Nyquist frequency of *f*_*Ny*_ ≈ 5.2 mHz. We performed the spectral analysis on linearly detrended data in a sliding window of 57 points, that is ≈91 min, corresponding to a Rayleigh frequency of *f*_*Ray*_ ≈ 0.18 mHz. To avoid border effects, we focused on the frequency range between ≈1.1 mHz and ≈4.1 mHz. For the spectral analysis of the one‐minute GOES observations at the geostationary orbit, we considered a sliding 91 point window. With a cadence of ≈60 s, the Nyquist frequency was *f*_*Ny*_ ≈8.3 mHz and the nominal frequency range unaltered by border effects is ≈1.1–7.2 mHz. At each step, we used the mean field evaluated on the entire interval to rotate the three components of the magnetic field into the Mean Field Aligned (MFA) coordinate system (Takahashi et al., 1990) and avoid spurious effects from the rotation procedure (Di Matteo & Villante, 2018). For each interval, we then removed the linear trend from the data before performing the spectral analysis.

We also investigated the polarization pattern of the detected waves using observations at ground stations. We performed a multitaper cross‐spectral analysis between the *B*_*N*_ and *B*_*E*_ components (*NW* = 3 and *K* = 4 as above) and applied the technique for partially polarized waves (Fowler et al., 1967). For waves at which the ratio between the polarized and total intensity of the horizontal signal was greater than 0.8, we estimated the azimuthal wave angle, formed by the major axis of the polarization ellipse and the northward direction, and the ellipticity, *ϵ*, that is the ratio between the minor and major axes of the polarization ellipse. Looking along the direction of the magnetic field, a positive ellipticity value corresponds to the right‐hand sense of rotation, while a negative value corresponds to the left‐hand sense of rotation. Here, we considered a wave right‐handed if *ϵ* > 0.2, left‐handed if *ϵ* < − 0.2, and linearly polarized otherwise. We also estimated the azimuthal wave number, *m*, from ground observatories in which a wave at a specific frequency was detected. We selected the station pairs separated by less than 1.5° in latitude and between 5° and 30° in longitude. Then, the azimuthal wave number is estimated as *m* = Δ*φ*/ΔΦ with uncertainty Δ*m* = 360Δ*t*/(*T*Δ*ϕ*) in which Δ*φ* is the phase difference of signals along one magnetic component between stations pairs, ΔΦ the stations' longitudinal separation, Δ*t* is the timing error considered as half the sampling time (30 s), and *T* is the period of the wave under investigation (Mathie & Mann, 2000). We estimated *m* along the *B*_*N*_ component for ground observatory pair below 60°, to avoid possible phase differences due to FLRs, and along the *B*_*E*_ component for ground observatory pair below 70°.

## 3 Event Overview

**Figure 1:**
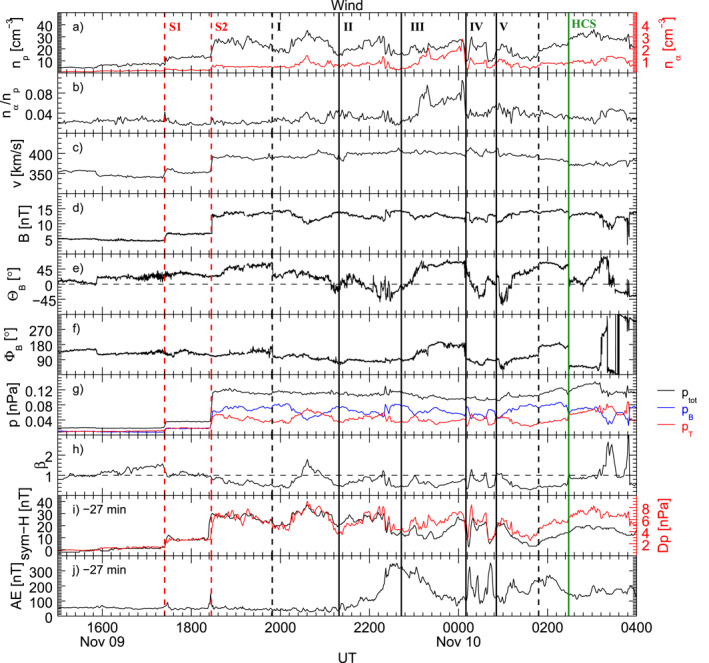
Solar wind parameters between 15:00 UT on 9 November 2002, and 04:00 UT on 10 November 2002, observed by Wind. From the top: proton and alpha number density; alpha to proton ratio; velocity; interplanetary magnetic field intensity and direction in geocentric solar magnetospheric coordinates; thermal, magnetic, and total pressure; plasma *β*; comparison of the solar wind dynamic pressure with the sym‐H index; AE index. Both the sym–H and AE index are shifted back in time by ≈27 min. The transit of two subsequent interplanetary shocks is marked by the red dashed lines. The black dashed lines delimit the time interval in which we identify ≈90 min periodic density structures delimited by the black dotted lines. The green dashed line marks the beginning of the transit through the heliospheric current sheet.

On 9–10 November 2002, a complex interplanetary structure impacted the magnetosphere. Figure 1 shows the solar wind parameters as observed by the Wind spacecraft located at *X*_*GSE*_ = 96.7 Re, Y_*GSE*_ = −29.7 Re, and *Z*_*GSE*_ = 5.5 Re. Two consecutive interplanetary shocks (red dashed lines) were observed on 9 November 2002. The first shock (S1) at ≈17:24 UT was characterized by a moderate jump in proton density (Δ*n*_*p*_ ≈ 5.3 cm^−3^), solar wind velocity (Δ*v* ≈ 18.2 km/s), magnetic field intensity (Δ*B* ≈ 2.3 nT), and dynamic pressure (Δ*Dp* ≈ 1.3 nPa). From the Interplanetary Shock Database by the Harvard‐Smithsonian Center for Astrophysics (http://www.cfa.harvard.edu/shocks), according to the Rankine‐Hugoniot relations, this discontinuity was a fast forward shock moving with a speed of *v*_*sh1*_ ≈ 381 km/s in the direction Φ_*sh1*,*GSE*_ ≈ 173.5° and Θ_*sh1*,*GSE*_ ≈ −0.3°. Following S1, all the solar wind parameters remained almost constant with no large amplitude fluctuations up to the transit of the second shock (S2) at ≈ 18:27 UT when we observed a jump in proton density (Δ*n*_*p*_ ≈ 13.4 cm^−3^), solar wind velocity (Δ*v* ≈ 36.5 km/s), magnetic field intensity (Δ*B* ≈ 4.0 nT), and dynamic pressure (Δ*Dp* ≈ 5.8 nPa) of larger amplitude with respect to S1. According to the Rankine‐Hugoniot relations, this was a fast forward shock moving with a speed of *v*_*sh2*_ ≈ 425 km/s in the direction Φ_*sh2*,*GSE*_ ≈ 181.5° and Θ_*sh2*,*GSE*_ ≈ −11.3°.

After ≈82 min from S2, Wind observed strong fluctuations in *n*_*α*_/*n*_*p*_ for ≈6 hr, bounded by rapid variations of the magnetic field direction detected at ≈19:49 UT on November 9 and at ≈01:48 UT on November 10 (vertical black dashed lines). The solar wind velocity was ≈398 km/s and showed very small variations. Within this time interval, we identified five *n*_*p*_ enhancements, delimited by the vertical dotted lines. Applying our spectral analysis procedure on the density observation for the entire interval, we identified a periodicity at ≈0.16–0.21 mHz (≈80–100 min) confirming the quasi‐periodic nature of these structures (Di Matteo et al., 2019; Kepko et al., 2016; Viall & Vourlidas, 2015). This paper focuses on the substructures and periodicities within each of these larger structures which hereby we refer to as: PDS I from ≈19:49 UT to ≈21:19 UT (≈90 min); PDS II to ≈22:43 UT (≈84 min); PDS III to ≈00:10 UT (≈87 min); PDS IV to ≈00:51 UT (≈41 min); PDS V to ≈01:46 UT (≈57 min).

The PDS I exhibited a peak of ≈35.4 cm^−3^, associated with an increase in *n*_*α*_ peaking at ≈1.44 cm^−3^ with a consequent *n*_*α*_/*n*_*p*_ of ≈0.04. At the same time, Wind observed a dip in the magnetic field intensity and increase of the plasma beta (*β* ≈ 1.8). In panel g, the anti‐correlation between the thermal and magnetic pressure was associated with very low variations of the total pressure indicating that this solar wind parcel was in pressure balance. Between PDSs I and II, the IMF slightly turned southward while *n*_*α*_/*n*_*p*_ fluctuated around 0.038. The PDS II was characterized by smaller‐scale density fluctuations whose boundaries were related to the rapid variation of the IMF direction (mostly *Θ*_*B*_). Variations in *n*_*p*_ and *n*_*α*_/*n*_*p*_ were correlated and peak values were associated with *β* ≈ 1. The substructures were in pressure balance, as evident from the almost constant total pressure, except at ≈22:21 UT when Wind observed a pulse in the total pressure, associated with a jump in the IMF intensity, at the boundary between two consecutive substructures. The PDS III exhibited *n*_*p*_ fluctuations at smaller scales as well. After an initial density enhancement during which the IMF turned northward and the plasma *β* peaked at unity, Wind observed a large increase in *n*_*α*_/*n*_*p*_ reaching values as high as ≈0.10. The PDS IV, confined by strong dips in *n*_*p*_, showed similar small scales fluctuations in *n*_*p*_ and *n*_*α*_. During this interval, the solar wind velocity and IMF intensity manifested a stronger variation with respect to the surrounding plasma, but the almost constant total pressure indicated that the structure was in pressure balance. The PDS V was also characterized by very similar fluctuations in *n*_*p*_ and *n*_*α*_. In addition, the first density increase was associated with a southward IMF and *β* ≈ 1. Following the periodic density structures, the polarity of the interplanetary magnetic field changed marking the beginning of the spacecraft transit through the HCS. Starting from a sharp rotation of the IMF on November 10 at ≈02:28 UT (vertical green dash‐dotted line), we noted an increase in *n*_*p*_, a decrease of the solar wind velocity, stronger dips in the IMF intensity, an increase in the total pressure, and plasma *β* close to or greater than one.

**Figure 2:**
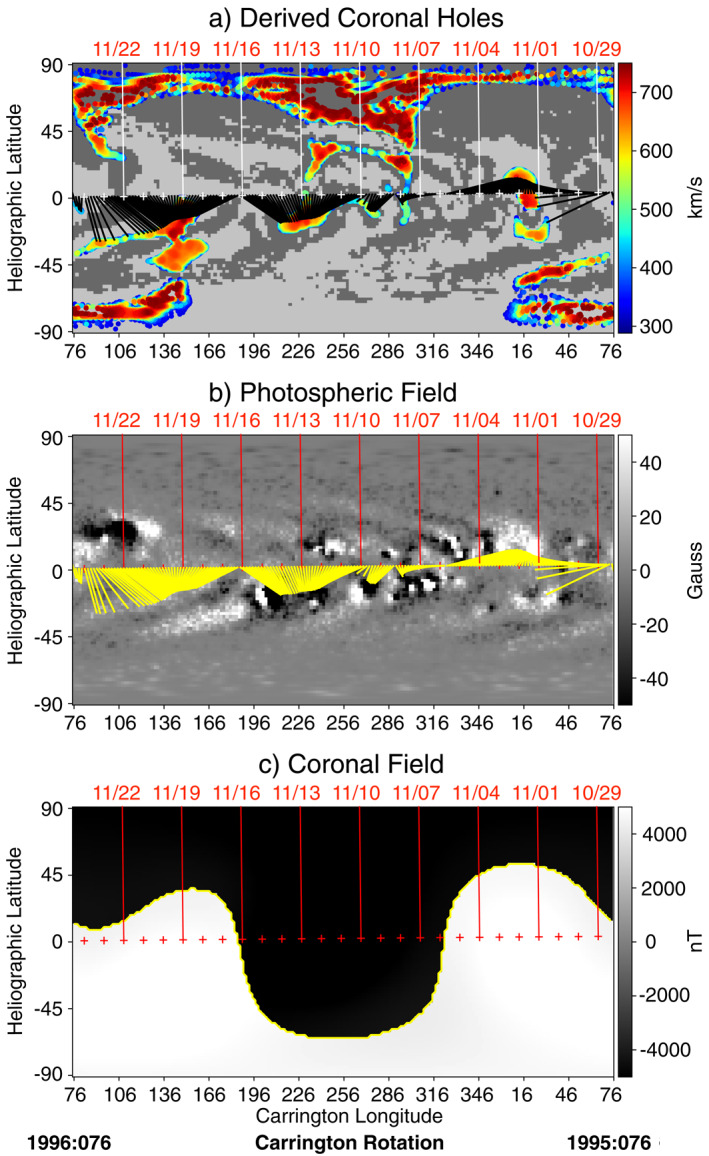
Wang‐Sheeley‐Arge (WSA) model output for CR 1995–1996 (29 October – 24 November 2002) derived from Air Force Data Assimilative Photospheric Flux Transport‐Kitt Peak Vacuum Telescope (ADAPT‐KPVT) input photospheric field maps. White (a) or red (b),(c) tick‐marks label the sub‐satellite points, representing the back‐projection of Wind's location at 5 *R*_⊙_ with dates labeled above in red. (a) WSA‐derived open field at 1 *R*_⊙_ with model‐derived solar wind speed in color scale. The field polarity at the photosphere is indicated by the light/dark (positive/negative) gray contours. Black lines show the magnetic connectivity between the projection of Wind's location at 5 *R*_⊙_ and solar wind source region at 1 *R*_⊙_. (b) Synchronic ADAPT‐KPVT photospheric field for 10 November 2002 20:00:00 UTC, which reflects the timestamp of the last magnetogram assimilated into this map. (c) WSA‐derived coronal field at 5 *R*_⊙_. Yellow contour marks the model‐derived heliospheric current sheet, where the overall coronal field changes sign.

We also used the WSA model to identify the source region of this solar wind stream, shown in Figure 2a. This event occurred during Carrington rotation (CR) 1996 (≈3 November – 30 November 2002). In Figure 2, the projection of Wind's location at 5 *R*_⊙_ is represented by the white/red cross hairs. The dates in Figure 2 correspond to when the solar wind left the Sun as opposed to when it arrived at Wind. The source regions of the solar wind observed at Wind is determined by tracing the WSA solution from 5 *R*_⊙_ to 1 *R*_⊙_ (black/yellow lines in Figures 2a and 2b respectively). According to the model solution, this solar wind stream left the Sun on ≈6 November 2002, emerging from an active region and a mid‐latitude coronal hole of positive polarity (≈16° Carrington longitude) up until Wind crosses the HCS (≈320° Carrington longitude). After the HCS (yellow line in Figure 2c) crossing, the solar wind emerged from another active region and mid‐latitude coronal hole (negative polarity) extending from the northern polar coronal hole (≈285° Carrington longitude). The WSA model‐derived IMF polarity and solar wind speed matched well with that observed at Wind, giving us high confidence in the source region identification.

**Figure 3:**
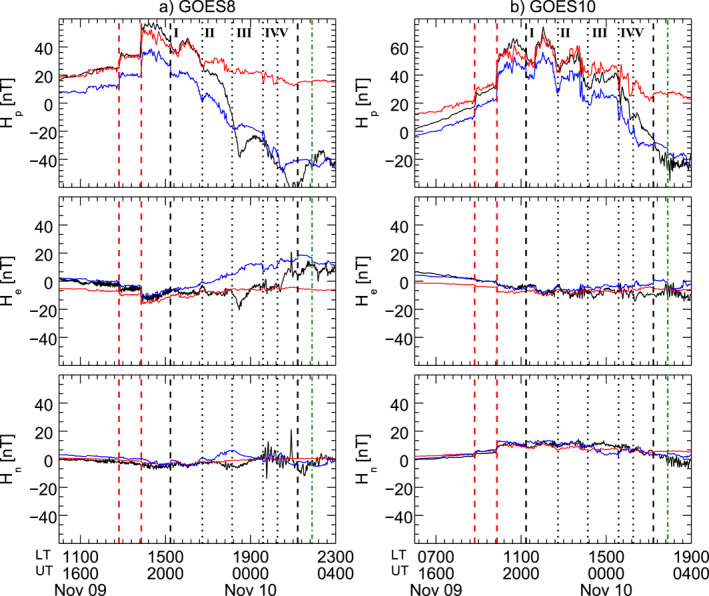
GOES8 (left panels) and GOES10 (right panels). The black lines show the magnetospheric field *H*_*p*_ (upper panels), *H*_*e*_ (middle panels), and *H*_*n*_ (lower panels) components at the geostationary orbit. The red and the blue lines show respectively the magnetic field predictions by the T04 model based on Wind observations, as obtained considering only the magnetopause current and all the currents systems. The contribution of the International Geomagnetic Reference Field field has been removed. The vertical lines are the same as in Figure 1, shifted by 25 min forward with respect to the wind observations.

We investigated the magnetospheric response at geostationary orbit using the magnetic field components as observed by GOES8 and GOES10 in the ENP coordinate system (Figure 3). Note that we removed the contribution of the long‐term variations by subtracting the International Geomagnetic Reference Field (IGRF; Thébault et al., 2015) at the satellite position. Based on Wind observations, the two interplanetary shocks were expected to impact the magnetosphere respectively after ≈28 and ≈24 min, that is at ≈17:52 UT and ≈18:51 UT. The corresponding Sudden Impulses (SI) were clearly observed at the geostationary orbit along the Hp component at ≈ 17:49 UT (GOES10 at ≈8:49 LT and GOES8 at ≈12:49 LT) and ≈18:48 UT (GOES10 at ≈9:48 LT and GOES8 at ≈13:48 LT), after ≈25 min and ≈21 min, in both cases 3 min before the expected time of impact. In Figure 3, we compared these observations with the prediction of the T04 model (Tsyganenko & Sitnov, 2005) based on the Wind observations considering the contribution of the magnetopause current only (*T*04_*MC*_; red lines) and all the currents system (*T*04_*all*_; blue lines). At both GOES satellites, the observed SIs are consistent with the ones expected for changes of the magnetopause current alone (Villante & Piersanti, 2008). The ground response at mid latitude magnetic observatories, represented by the sym‐H index (Figure 1i), showed the SIs at ≈17:51 UT and ≈18:50 UT, respectively, 2 min after the observations at the geostationary orbit. At higher latitudes, after additional 3 min, we observed a short amplification of the auroral electrojet as two peaks in the AE index (Figure 1j) of ≈94 nT and ≈150 nT at ≈17:54 UT and ≈18:53 UT.

After the impact of S2, the ≈90 min PDSs directly drove magnetospheric field fluctuations at the geostationary orbit along the *H*_*p*_ component. The observations of GOES8 and GOES10, in the dayside region, were well represented by the *T*04_*MC*_ model even at the smaller time scales. The observations deviate from the *T*04_*MC*_ model prediction, due to the effects of the tail and ring current, progressively from the end of the interaction with the PDS I for GOES8 at ≈21:44 UT (≈16:44 LT) and the PDS II for GOES10 at ≈23:08 UT (≈14:08 LT). Nevertheless, the small‐scale variations continued to correspond well with the *T*04_*MC*_ model. Therefore, the PDSs were associated with solar wind dynamic pressure variations which directly drove magnetospheric field fluctuations in the Pc5 frequency range. At mid and low latitude ground observatories, the magnetic field along the north‐south direction, represented by the sym‐H index showed in Figure 1i, closely follow the variation of the solar wind dynamic pressure (red line), approximately until the end of the interaction with the PDS II, similarly to GOES10. The AE index remained low for 3 hrs after the impact of S2 but started to increase, reaching a maximum of ≈350 nT, following a short period of southward interplanetary magnetic field (Figure 1e).

## 4 Spectral Analysis of Solar Wind and Magnetospheric Field Fluctuations

At geostationary orbit, in addition to the fluctuations that were directly correlated with changes in the solar wind, there were also evident fluctuations along the *H*_*e*_ and *H*_*n*_ component for both GOES satellites with no counterpart in the solar wind. Therefore, to better characterize the fluctuations in the Pc5 frequency range in the solar wind and in the magnetosphere, we performed a spectral analysis according to a novel procedure based on the multitaper method (Di Matteo et al., 2020, 2021) that is able to separate the continuous portion of the power spectral density from narrow and broad enhancements due to wave activity.

**Figure 4:**
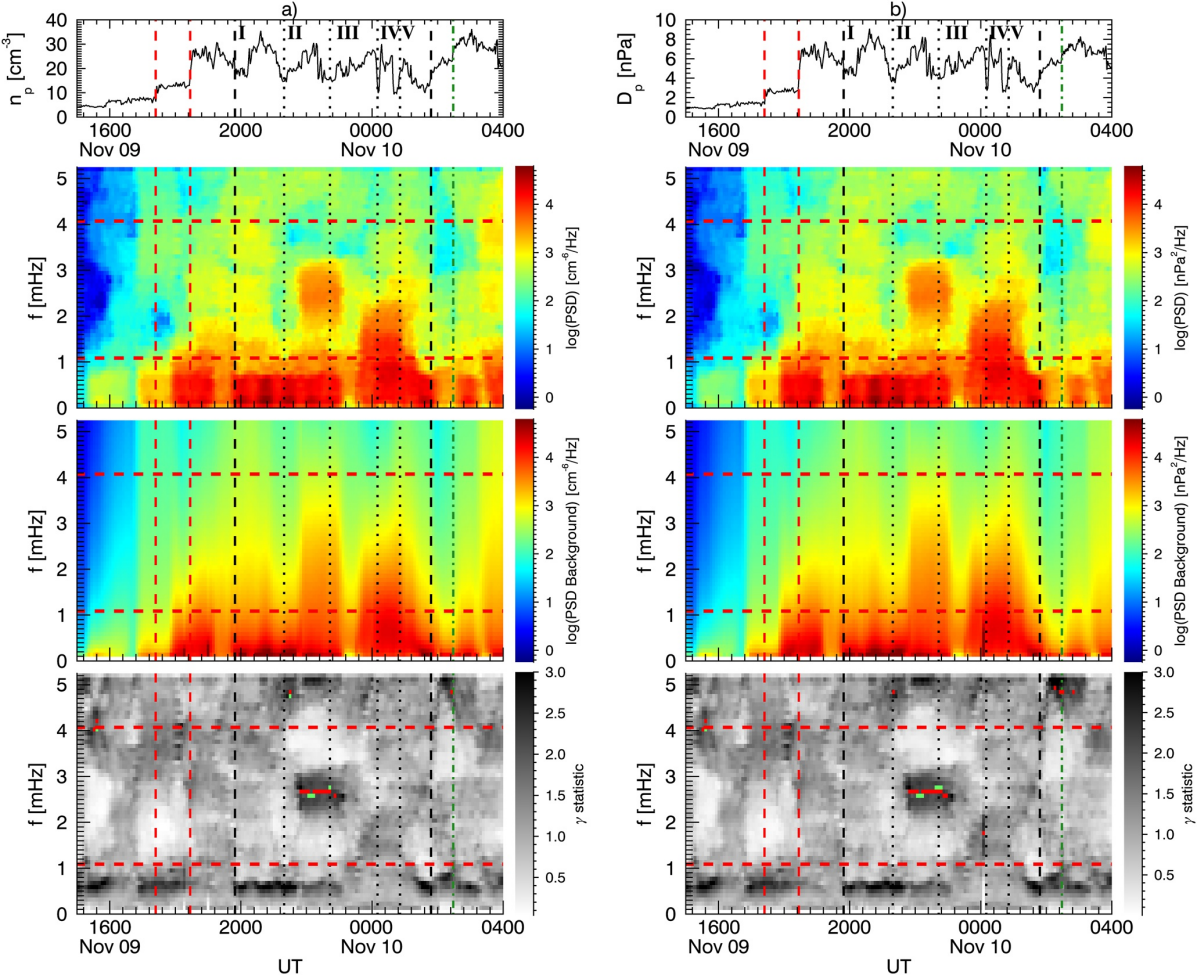
Spectral analysis of the solar wind proton density (panel a) and dynamic pressure (panel b) as measured by Wind. From the top, we show the time series, the dynamic spectrum, the estimated continuous background spectrum, and their ratio named *γ* statistic. The horizontal red lines delimit the frequency range free from higher rates of false positives, while the vertical lines are the same as in Figure 1. The red dots in the bottom panel identify the time and the center frequency of the power enhancements above the 90% confidence threshold (*γ* test). Within these intervals, the green dots mark the portions simultaneously passing the *F* test.

Figure 4 shows the spectral analysis results for the solar wind proton density and dynamic pressure. For each parameter, we show the time series, the dynamic spectrum, the estimated background spectrum, and their ratio termed *γ* statistic. In each panel, the horizontal red lines delimit the frequency range free from higher rates of false positives (see Section 2), while the vertical lines are the same as in Figure 1. The solar wind velocity showed little variation during this time interval so the dynamic pressure variations are entirely due to the solar wind density. This is confirmed by the practically identical results for the two parameters shown in Figure 4. An isolated power enhancement between ≈21:46 UT and ≈22:44 UT, centered at ≈2.6 mHz, passed the 90% confidence threshold of the *γ* test (red dots in bottom panels). Within the same time interval, the *F*‐test (green dots) further distinguished two signals at ≈ 2.5 mHz and ≈2.7 mHz, respectively, around ≈22:05 UT and ≈22:39 UT.

**Figure 5:**
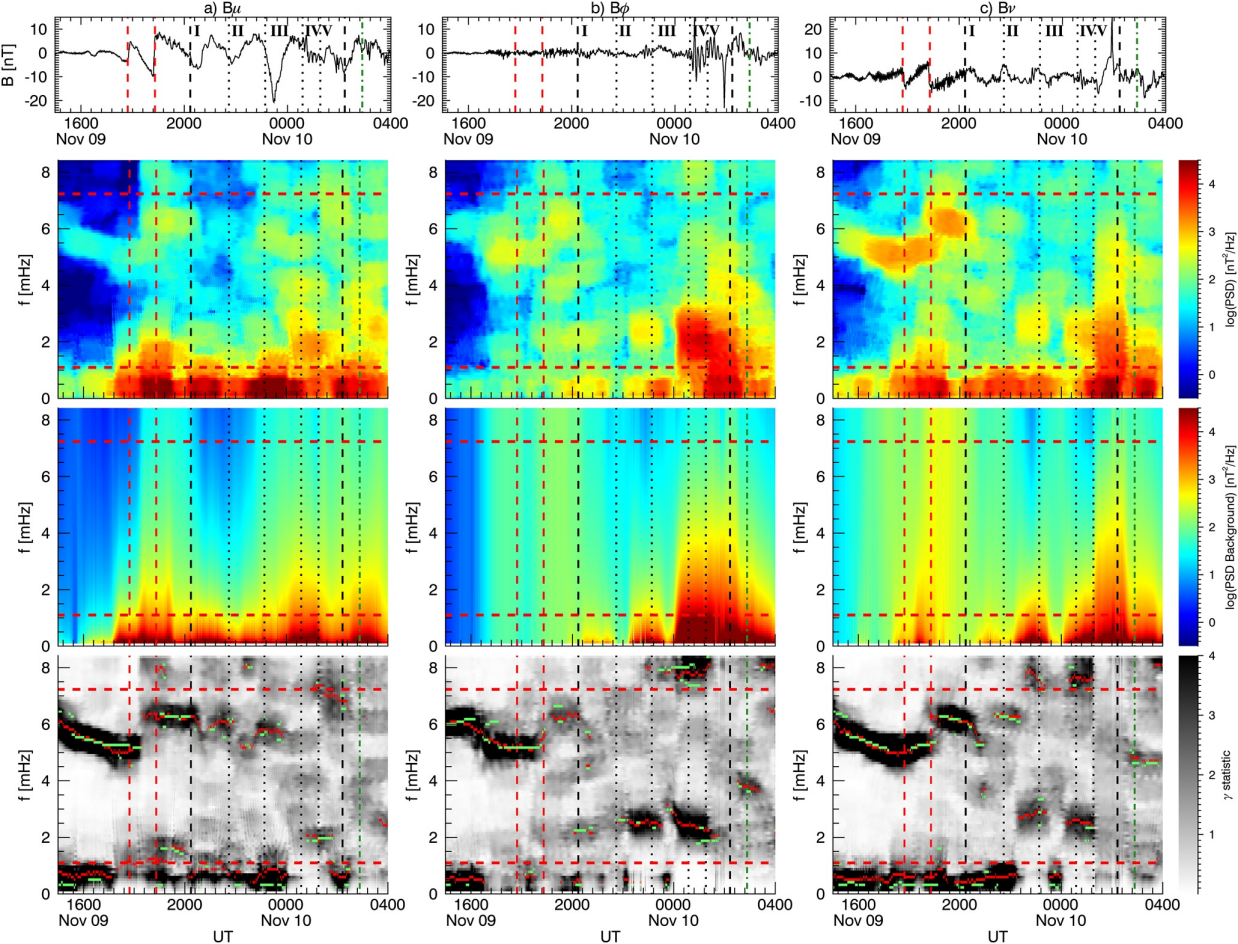
Dynamic power spectra of the magnetospheric field components in the MFA coordinate system at GOES8, as in Figure 4. From the left, the compressional (*B*_*μ*_), toroidal (*B*_*ϕ*_), and poloidal (*B*_*ν*_) components. The vertical lines are the ones in Figure 1 shifted of 25 min forward.

Figure 5 shows the spectral analysis results for the compressional (*B*_*μ*_), toroidal (*B*_*ϕ*_), and poloidal (*B*_*ν*_) magnetic field component at GOES8 with the same format used for the solar wind parameters. In the following, we refer to the results from the *γ*+F test (green dots in bottom panels) unless otherwise noted. After the impact of the second interplanetary shock, we observed a clear wave at ≈1.6 mHz along *B*_*μ*_, less evident along *B*_*ν*_. At the impact of the PDS I, we identified waves at ≈2.3 mHz and ≈4.5 mHz along *B*_*ϕ*_ and at ≈3.6 mHz along *B*_*ν*_. At the PDS II, the *γ* test revealed a clear power peak centered at ≈2.6 mHz along *B*_*ϕ*_. The *B*_*ν*_ component shows similar results but with the *γ*+F test marking three frequencies at ≈2.5, ≈3.0, and ≈3.4 mHz at the boundary with the PDS III. During the impact of the PDSs III‐IV‐V, we observed a broad power enhancement centered at ≈2.5 mHz, more evident for the *B*_*ϕ*_ component. The *F* test selected a wave at ≈1.9 mHz along both the *B*_*μ*_ and *B*_*ϕ*_ components and at ≈2.4 mHz along *B*_*ϕ*_ and *B*_*ν*_. At higher frequencies, we observed a clear wave activity lasting from the beginning of the time interval to ≈23:50 UT (≈18:50 LT). The wave frequency decreased from ≈6.2 mHz to ≈5.2 mHz before the first SI, smoothly for *B*_*μ*_ and *B*_*ν*_ and in a more step‐like manner for *B*_*ϕ*_. Between the two SIs, we continuously observed the wave at ≈5.2 mHz along *B*_*ϕ*_ and *B*_*ν*_. After the second SI, the wave frequency jumped to ≈6.4 mHz and appeared stronger on the *B*_*μ*_ and *B*_*ν*_ components. After the impact of the first PDS, the wave frequency varied seemingly following the solar wind dynamic pressure variations.

**Figure 6:**
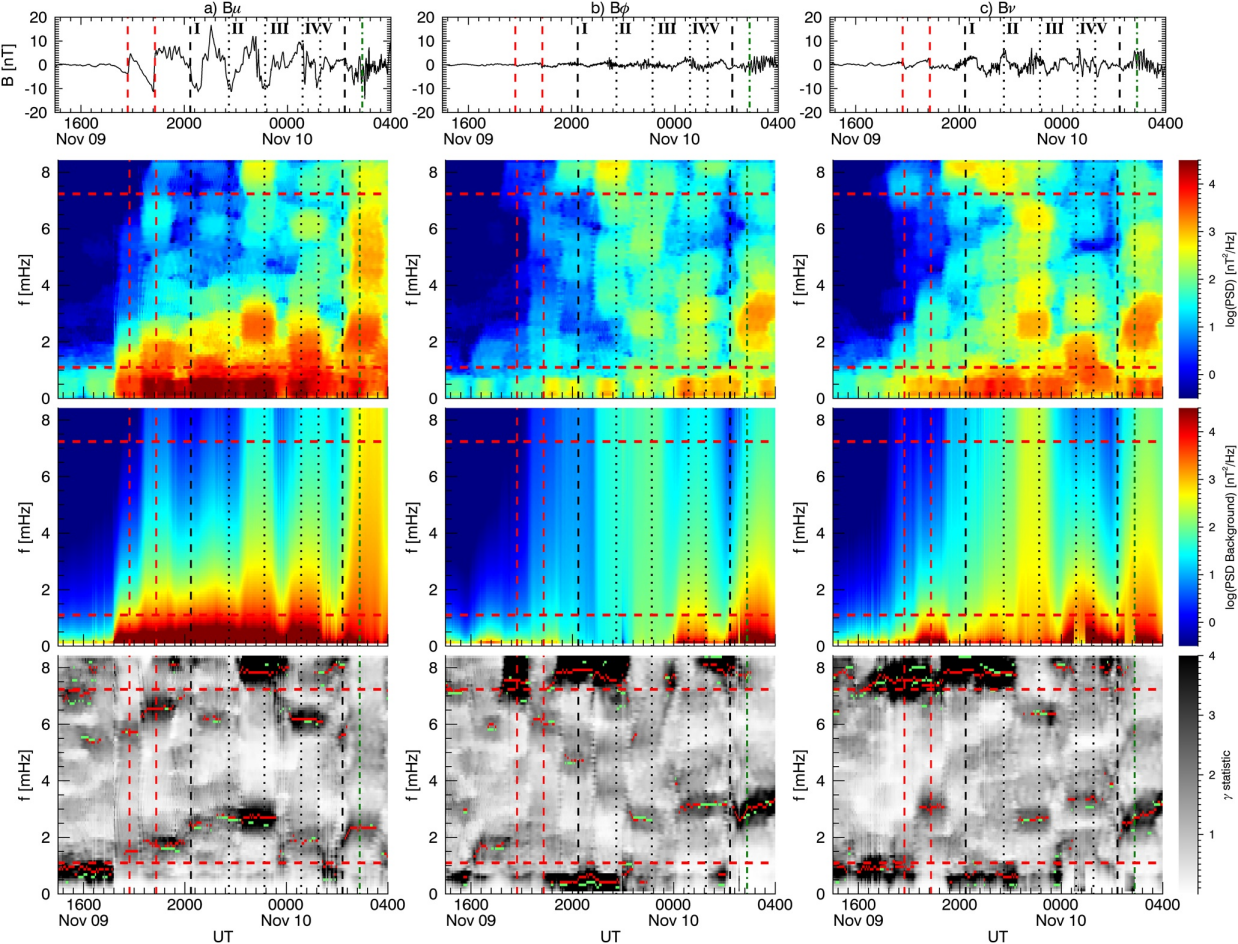
Same as Figure 5, with the magnetospheric field components in the mean field aligned coordinate system at GOES10.

We repeated the spectral analysis in the same format for GOES10 (Figure 6). After the impact of the second interplanetary shock, we observed a clear wave at ≈1.6 mHz along *B*_*μ*_ lasting for about 1 hr. Then, during the impact of the PDS I, we observed fluctuations at ≈2.4 mHz and ≈2.7 mHz, respectively at the beginning and the end of the interval. The latter persisted through the interaction with the PDS II and was detected also along *B*_*ϕ*_ and *B*_*ν*_. During the interaction with the PDSs III‐IV‐V, we observed a clear broad power enhancement between 1 and 2 mHz along *B*_*μ*_ and *B*_*ν*_ corresponding to a portion of the time series that clearly resemble the solar wind dynamic pressure profile. However, the *γ*+F test (green dots in the bottom panels) selected a wave only along the *B*_*μ*_ component at ≈1.9 mHz. Along *B*_*ϕ*_ and *B*_*ν*_ instead, the *γ*+F test revealed evidence of a wave at ≈3.2 mHz. At higher frequency, there was no clear correspondence with the wave observed at GOES8. We identified only short power enhancements at ≈5.6 mHz on *B*_*μ*_ and ≈5.9 mHz on *B*_*ϕ*_ before the first SI; at ≈6.2 mHz on *B*_*μ*_ during the PDSs I and IV; and at ≈6.5 mHz *B*_*ν*_ between the PDSs II and III. Note that, unlike the observations at GOES8, the power peaks centered at the SIs are isolated and can be artifacts due to the jump in the time series. Finally, we noted a possible strong wave activity at a frequency above ≈7.0 mHz, mostly along *B*_*ν*_. However, this interval is outside the reliable frequency range of our methodology.

## 5 Response at Ground Magnetometers

**Figure 7:**
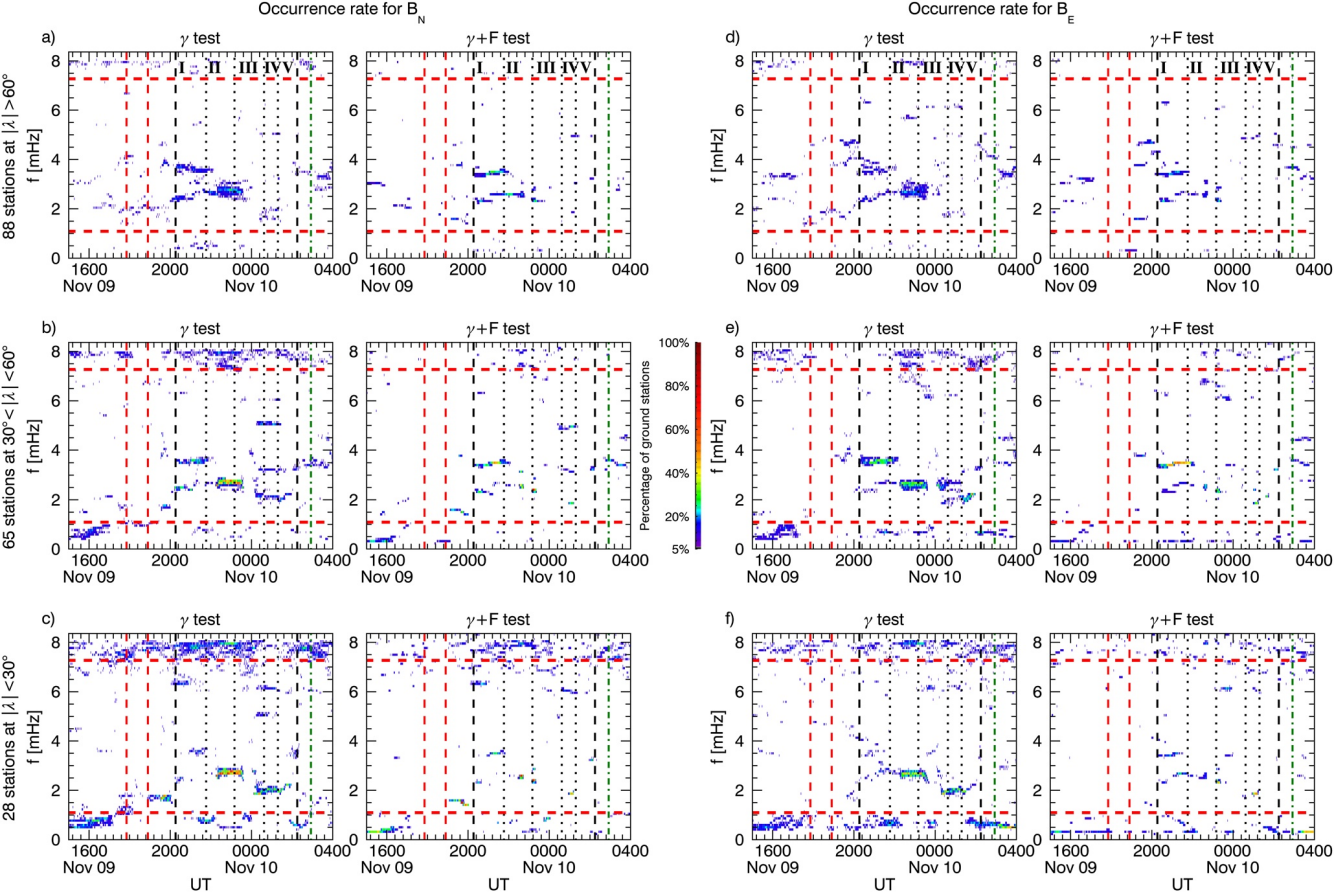
The percentage of ground observatories in which we identified a wave at a specific frequency according to the *γ* and the *γ* + F test. From the top, the occurrence rate for high, mid, and low latitude stations respectively for the *B*_*N*_ (panel a–c) and the *B*_*E*_ (panel d–f) components. The horizontal red lines delimit the frequency range free from higher rates of false positives, while the vertical lines are the ones in Figure 1 shifted 27 min forward.

**Figure 8:**
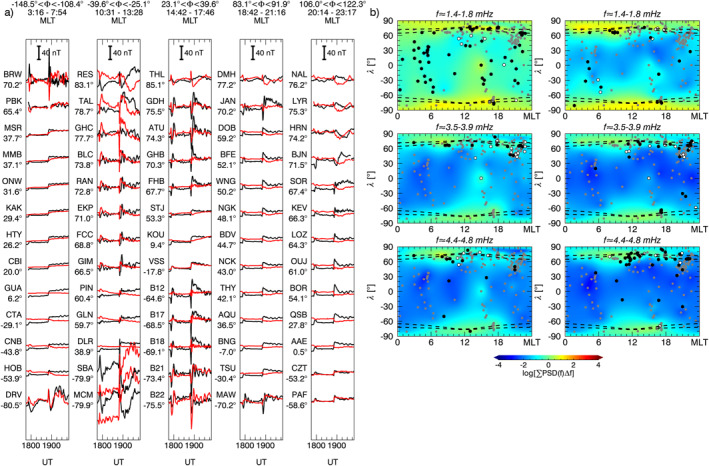
Panel a, stackplot of the *B*_*N*_ (black) and *B*_*E*_ (red) component time series for five latitudinal ground observatories arrays. Panel b, for a ≈91 min time interval centered at ≈19:52 UT on 9 November 2002, global maps of the integrated power spectrum on ≈0.54 mHz frequency intervals centered at ≈1.5, ≈3.7, and ≈4.6 mHz, for the *B*_*N*_ (left) and *B*_*E*_ (right) components. At the locations of the ground observatories used for the analysis (gray dots), white and black dots indicate the identification of a wave with the *γ* and *γ*+F test, respectively, within 10 min from the map time. The dashed lines represent the auroral oval boundaries.

**Figure 9:**
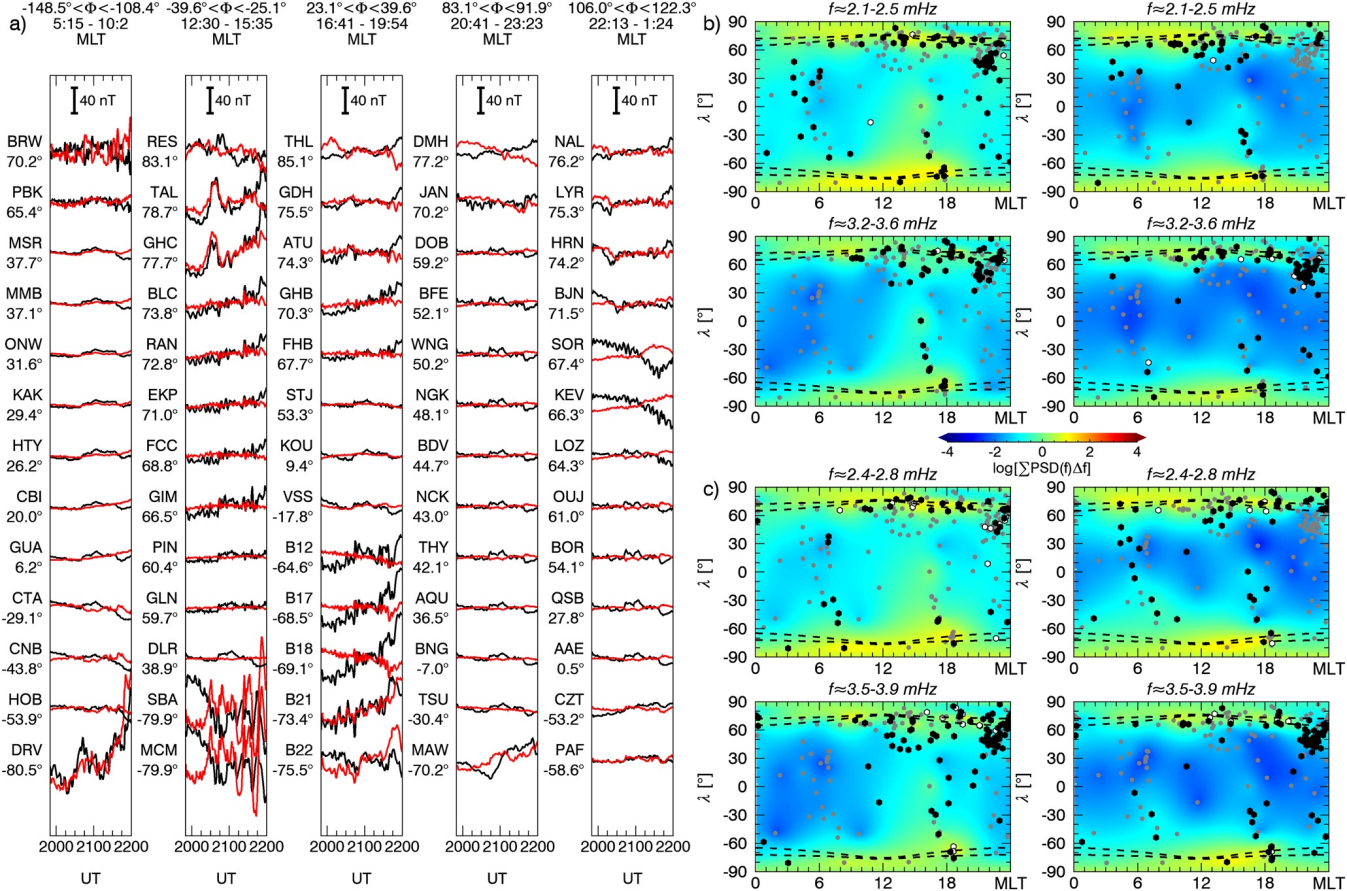
Same as Figure 8 with global maps of the integrated power spectrum for a time interval centered at ≈20:30 UT and frequency intervals centered at ≈2.3, and ≈3.4 mHz (panel b). Panel c, the same as panel b for an interval centered at ≈21:20 UT and frequency intervals centered at ≈2.6, and ≈3.7 mHz.

**Figure 10:**
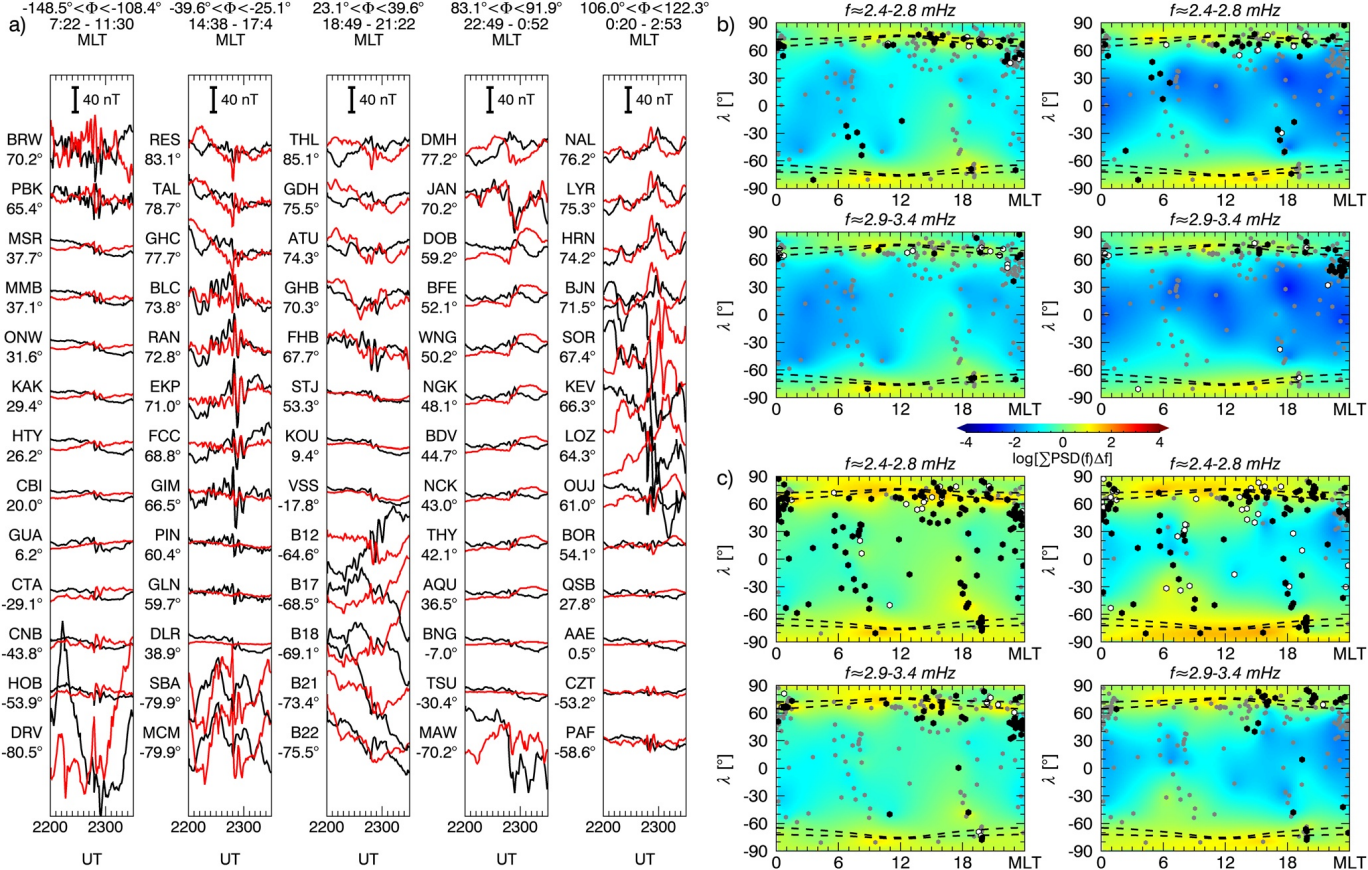
Same as Figure 8 with global maps of the integrated power spectrum for a time interval centered at ≈21:50 UT and frequency intervals centered at ≈2.6, and ≈3.7 mHz (panel b). Panel c, the same as panel b for an interval centered at ≈22:35 UT and centered at the same frequency intervals.

**Figure 11:**
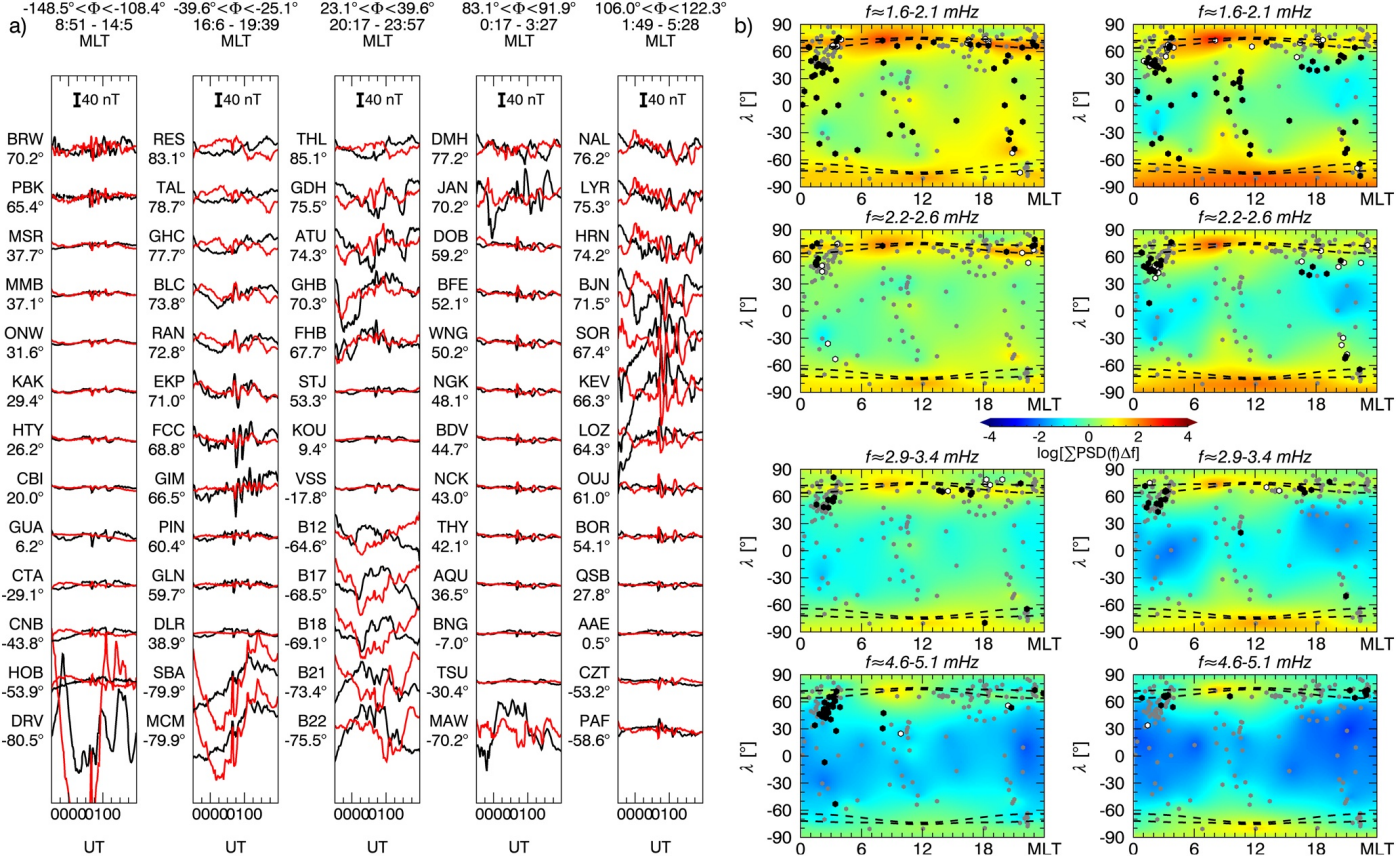
Same as Figure 8 with global maps of the integrated power spectrum for a time interval centered at ≈01:03 UT on 10 November 2002, and frequency intervals centered at ≈1.8, ≈2.4, ≈3.1, and ≈4.9 mHz.

We continued our analysis considering the one–minute magnetic field measurements from 181 ground observatories available from the SuperMAG collaboration. Using the same parameters as in the previous section, we applied our spectral analysis procedure on the *B*_*N*_ and *B*_*E*_ magnetic field components. For each observatory, we collected the portion of the dynamic spectrum passing the *γ* test and the *γ*+F test at the 90% confidence level. We show the results of the spectral analysis in Figure 7 for both the *B*_*N*_ (left panels) and *B*_*E*_ (right panels) component at stations divided into three groups by magnetic latitude: high (*λ* > 60°, panel a and d), mid (30° < *λ* < 60°, panel b and e), and low latitude (*λ* < 30°, panel c and *f*). The color scale indicates the percentage of stations that detected a wave at a specific frequency and time according to the *γ* test and the *γ*+F test. We noted that the *γ* test results spread over a wider frequency range, especially at higher latitudes; however, the combination with the *F*‐test drastically reduces this effect allowing finer analysis. Therefore, in the following discussion, the results pertain the outcome of the *γ*+F test, unless otherwise noted. In addition, to better present the global response at the ground for each time interval, we show in Figures 8, 9, 10, 11 a stack‐plot for the *B*_*N*_ (black) and *B*_*E*_ (red) component at selected ground observatories in four magnetic longitude (Φ) sectors. In each Figure, we also show a qualitative representation of the global power distribution for *B*_*N*_ and *B*_*E*_ relative to a ≈91 min interval centered at specific times. We integrated the power spectral densities over a frequency range derived extending the frequencies identified by ≈0.27 mHz on both sides. Then, we interpolated the scattered power values to a regular grid using the Kriging method (Isaaks & Srivastava, 1989). In each map, the gray dots represent the ground observatories' position; the white and black dots indicate respectively the stations for which the *γ* and the *γ*+F test passed the 90% confidence threshold in any moment between 10 min before and after the map time. For context, we also included the auroral zones position (Holzworth & Meng, 1975). In the next four sections, we describe the entire response of the magnetosphere as a function of time, separated by the larger solar wind features.

### 5.1 ULF Wave Response to the Impact of S1 and S2: 17:45‐19:50 UT

At ground (Figure 8a), we observed globally the clear signature of the shocks impact as a SI at the mid and low latitude and a double pulse at high latitude (Araki, 1994; Piersanti & Villante, 2016). The short length of the time interval between the two SIs prevented a robust spectral analysis since it would be affected by the jumps in the time series. However, the stack‐plot of the ground magnetic field in Figure 8a shows, after the impact of S1, a strongly damped ULF wave (C. Wang et al., 2015) at ≈1.9 mHz along the *B*_*N*_ component approximately in the ≈10:00‐20:00 MLT sector at 66° ≲ |*λ*| < ≲74°. Fluctuations at ≈3 mHz occurred in the ≈14:45‐15:45 MLT sector at 65° ≲ |*λ*| ≲76°. No clear wave response was observed at mid and low latitudes.

After the second SI (Figure 7), we detected waves at ≈1.5 mHz along the *B*_*N*_ component at low and mid latitude stations, while at high latitude we obtained lower rates in both *B*_*N*_ and *B*_*E*_. At high latitude stations, we detected waves at ≈3.7 and ≈4.6 mHz with higher rates along the *B*_*E*_ component; some trace of the ≈3.7 mHz wave was retained at mid latitudes, while we found no evidence at low latitudes. The response is better represented in the global distribution of power centered at ≈19:52 UT in Figure 8b for *B*_*N*_ (left) and *B*_*E*_ (right). Along the *B*_*N*_ component, the waves at ≈1.5 mHz were evident at all latitudes below the auroral zones in the ≈0–6 MLT sector and at latitudes between ≈−50° and ≈50° and along the auroral zones in the remaining MLT sector. Along the *B*_*E*_ component, the results are sparse with some evidence along the auroral oval latitudes and at low latitudes in the night‐side sector. The wave at ≈3.7 mHz was evident at latitudes between ≈60° and ≈70° at all MLT along *B*_*N*_, and for MLT >12 along *B*_*E*_. We also found some evidence at lower latitudes at ≈12 MLT and ≈21 MLT. In the southern hemisphere, we found clear evidence of the ≈3.7 mHz wave along *B*_*E*_ between the B12 and B18 ground stations, as can be also seen in the corresponding time series in Figure 8a. The wave at ≈4.6 mHz was detected along the *B*_*N*_ component in the ≈7–12 MLT sector at latitudes between ≈50° and ≈65°, and in the ≈12–16 MLT above ≈70°. Along the *B*_*E*_ component, the wave is observed mostly for MLT >10 down to a latitude of ≈50°. Note that sparse detection at latitudes |*λ*| < 30° associated with low power (dark blue areas in Figure 8b) are likely false positives. In summary, the magnetosphere exhibited different distributions and persistence of ULF wave response to the two shocks.

### 5.2 Response to the PDS I: 19:50‐22:00 UT

Immediately after the impact on the magnetosphere of the IMF discontinuity marking the beginning of the PDS I (first black dashed line in Figure 7), we observed waves at ≈2.3 and ≈3.4 mHz. The former suddenly jumped to ≈2.6 mHz in correspondence with an increase of the solar wind dynamic pressure, while the latter rose gradually reaching ≈3.7 mHz. These signatures were evident at high latitudes stations on both magnetic field components; at mid latitudes we detected the same waves but with higher rates for the ≈3.4/3.7 mHz, especially along the *B*_*E*_ component. At low latitudes, the waves were mostly detected along the *B*_*E*_ component; along the *B*_*N*_ component, we observed some relevant signature only at ≈3.7 mHz in the second half of the interval.

At low and mid latitude stations, there is a high correlation with the solar wind density for all MLTs (Figure 9a), while at high latitude stations and in the dusk sector we observed clear additional fluctuations. As with the previous interval, we show a global map of the waves power distribution and occurrence at ground for ≈91 min intervals centered at ≈20:30 UT (Figure 9b) and ≈21:20 UT (Figure 9c). The wave at ≈3.4 mHz manifested along the *B*_*E*_ component encompassing more ground observatories at mid latitude. On the other hand, along the *B*_*N*_ component we detected wave activity at mid and high latitude stations, mostly between 12 MLT and 24 MLT in the north hemisphere, and at all latitudes below the auroral oval between 15 MLT and 18 MLT in the south hemisphere. In the second half of the interval, the ≈2.3 mHz wave was replaced by one at ≈2.6 mHz, which manifested similar properties, while the ≈3.4 mHz slightly rose to ≈3.7 mHz. Comparing Figure 9c with Figure 9b, the ≈2.6 mHz wave along the *B*_*N*_ component faded at mid and low latitude, while persisting and intensified at high latitude. Along the *B*_*E*_ component, the wave occurred at a lower number of stations at high latitude and at a higher number at mid and low latitude in the dayside sector. For the ≈3.7 mHz wave, there was an overall increase in the number of observatories detecting the waves, mostly confined in the afternoon sector.

### 5.3 Response to the PDS II: 22:00‐23:30 UT

At the interaction with the PDS II, the wave at ≈3.7 mHz gradually faded everywhere while the one at ≈2.6 mHz persisted at high latitudes mostly along the *B*_*N*_ component (Figure 7a). In Figure 10b, we show that there is a clear similarity with the results in Figure 9c, but with the lower occurrence at mid and low latitude ground observatories. Later, the solar wind parcel showing clear PDSs at ≈2.6 mHz impacted the magnetosphere. The *γ* test results revealed a clear power spectrum enhancement in the ≈2.2–2.6 mHz frequency range at mid and low latitudes, involving almost all ground observatories, while at high latitudes the selected frequencies spread over a wider range. On the other hand, within the same interval the *γ*+F test selected waves at ≈2.6 mHz and ≈3.1 mHz, with the latter more evident at high latitudes stations. The occurrence of a strong broad power spectrum enhancement associated with multiple peaks in the *F* test is an expected result in the case of multiple signals with frequency separation smaller than the width of the main lobe of the spectral window (Di Matteo & Villante, 2017). Our methodology allows the clear distinction of waves at a frequency separated by more than half‐width of the main lobe, that is ≈ 0.55 mHz based on the choice of the spectral analysis parameters. The occurrence of these short periods with two selected waves might correspond to the time in which our technique was able to resolve them. Note that at the same time the interplanetary magnetic field turns southward and the AE index reached is maximum marking a substorm. This additional activity manifested in the ground ULF waves power distribution in Figure 10c as an intensification at high latitude. Even though our interpolation method is qualitative, the areas with enough ground observatories show that the wave power along the *B*_*N*_ component is confined in the auroral zones, closer to the equatorward boundary. Nevertheless, the PDSs directly drove a global ULF wave mode at ≈2.6 mHz. The associated fluctuations are clearly visible at all latitudes at the center of the time series, shown in Figure 10a, and are detected along both *B*_*N*_ and *B*_*E*_ (Figure 10c). Note that the directly driven wave was evident even in presence of ongoing wave activity at similar frequency (e.g., from GIM to BLC), substorm activity (e.g., from LOZ to SOR), and in polar cap stations (De Lauretis et al., 2016). The wave at ≈3.1 mHz remained confined in the afternoon sector mostly at mid and high latitudes, similarly to the higher frequency counterpart in the previous intervals. At the boundary between the PDS II and III, between 23:09 UT and 23:19 UT, the frequency of the waves moved toward slightly lower frequencies at ≈2.4 mHz and ≈2.9 mHz, but retained the same properties.

### 5.4 Response to the PDSs III‐IV‐V: 23:30‐02:00 UT

During the interaction of the PDSs III‐IV‐V (Figure 7), we identified a wave at ≈ 1.8 mHz at mid and low latitudes on both magnetic field components. Moving at a higher frequency, we noticed waves localized at mid latitude stations at ≈2.4 mHz along the *B*_*E*_ component, better recognized in the *γ* test, and at ≈3.1 mHz along the *B*_*N*_ component. Finally, we identified high occurrence rates at ≈4.9 mHz at low and mid latitude stations along the *B*_*N*_ component. The time series of the magnetic field at the ground in Figures 11a show the resemblance with the solar wind density profile at mid and low latitude stations. While the density variations in the solar wind are sharp and determined an overall power enhancement in the dynamic spectrum up to ≈2 mHz (Figure 4), at the ground the response is smoother and resulted in the global oscillations at ≈ 1.8 mHz. The corresponding integrated wave power distribution, for a ≈91 min interval centered at ≈01:03 UT on 10 November (Figure 11b), was higher than the previous intervals due to the substorm activity. Interestingly, the power along the *B*_*N*_ component in this frequency range matched nicely the auroral oval in the night‐side sector, where there was wide ground stations coverage. The ≈1.8 mHz wave was observed globally, but with preferential locations for the *B*_*N*_ and *B*_*E*_ components: along the former, we identified the wave well below the auroral oval, except in the 10–19 MLT sector where it was close to the equatorward auroral oval border; for the latter, we detected the wave mostly at mid latitude in the night‐side sector and at a low latitude in the 24‐12 MLT sector. The ≈2.4, ≈3.1, and ≈4.9 mHz manifested along both *B*_*N*_ and *B*_*E*_ components at mid and high latitudes in the 1–4 MLT sector, but we found some evidence also in the afternoon sector. Interestingly, the ≈2.4 mHz wave occurred along the auroral oval at ≈20–24 MLT along *B*_*N*_ and at mid latitudes at ≈16–22 MLT along *B*_*E*_. The ≈3.1 mHz wave was evident close to the equatorward auroral oval border at ≈13–17 MLT along both *B*_*N*_ and *B*_*E*_.

## 6 Electron Radiation Belt Response

We investigated the response of radiation belt electrons at six geostationary satellites analyzing spin‐averaged electron fluxes at an energy ranging from 50 keV to 6.0 MeV. Figure 14 shows the measurements for the entire interval in analysis. Here, we focus on the response to the clear monochromatic solar wind PDSs, namely the 0.18 mHz (≈90 min) and the 2.6 mHz (≈6.4 min).

**Figure 12:**
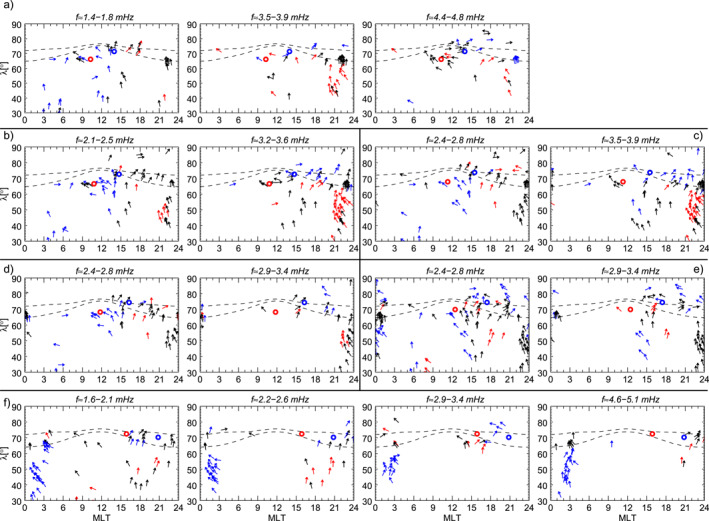
Polarization analysis for ground observatories in the northern hemisphere (*λ* > 30°) detecting a wave in the same frequency and time intervals used in Figure 8 (panel a), Figure 9 (panel b–c), Figure 10 (panel d–e), Figure 11 (panel f). At the location of each ground observatory, when the degree of polarization is greater than 0.8, the arrows indicate the direction of the major axis of the polarization ellipse. Red, blue, and black arrows represent right‐handed, left‐handed, and linear polarization, respectively. The red and blue circles represent the footpoint of the magnetic field line passing respectively through GOES8 and GOES10 using the T04 model. The dashed lines represent the auroral oval boundaries.

**Figure 13:**
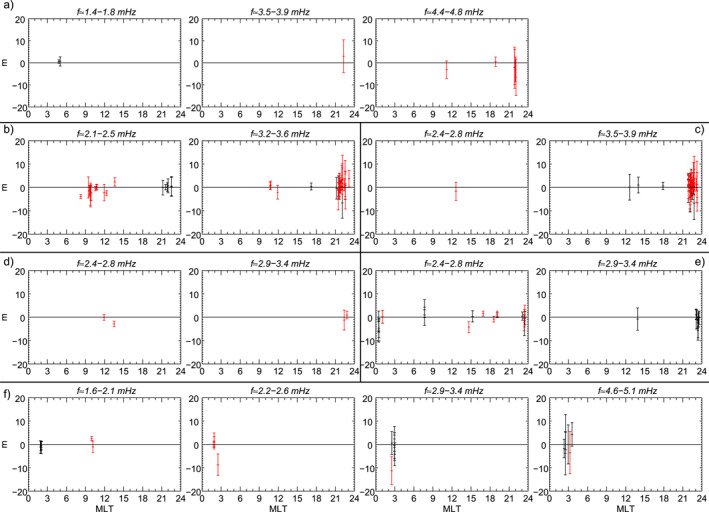
Azimuthal wave number estimated from ground observatories pairs detecting a wave in the same frequency and time intervals used in Figure 8 (panel a), Figure 9 (panel b–c), Figure 10 (panel d–e), Figure 11 (panel f). Black and red indicate estimates obtained respectively from the *B*_*N*_ component, for stations at *λ* < 60°, and *B*_*E*_ component, for stations at *λ* < 70°.

**Figure 14:**
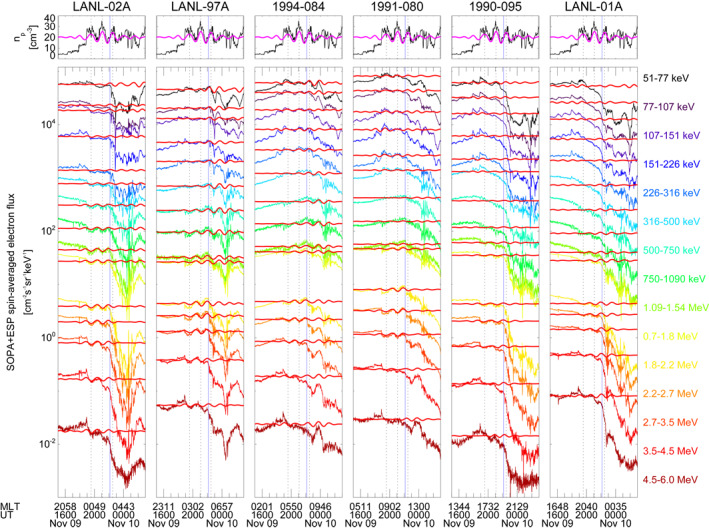
One‐minute electron particle flux data at the geostationary orbit for 15 differential energy channels from six Los Alamos National Laboratory satellites compared with the solar wind proton density (top panels) for the entire time interval in analysis. Magenta and red lines show the observation filtered in the 0.15–0.25 mHz frequency range. The vertical lines identify amplitude peaks for the 90 min periodic density structures. The blue vertical line identifies the substorm onset at 22:08 UT.

At all satellites, the sharp variations occurring at the impact of the two interplanetary shocks and the rapid decrease following the substorm onset at 22:08 UT (Ohtani & Gjerloev, 2020) prevented a robust spectral analysis for the identification of the 90 min periodicity. Therefore, to better follow the periodic fluctuations, we show the filtered Wind (LANL) observations (magenta and red lines in Figure 14) in the 0.15–0.25 mHz (≈67–111 min) frequency range obtained with a Kaiser window filter of length 293 (487) points with stopband gain of −50 dB (Oppenheim et al., 1999). The 1991‐080 satellite, closest to noon, observed prompt coherent flux enhancements for electron energies ranging from 50 to 500 keV in response to the 90 min PDS, identified by the vertical dotted lines, with similarities even at smaller timescales resembling the waves following the two shocks and the PDS I density substructures. Moving away from noon, the modulation was retained only at longer time scales and for progressively lower energy. Interestingly, in the post‐midnight sector (LANL‐02A) we observed the 90 min modulation in antiphase with respect to the solar wind variations for fluxes at energies above 107 keV. This effect was observed globally but pertaining a narrower energy range reaching its minimum at noon (1991‐080) where the modulation was evident for fluxes at energies greater than 1 MeV.

**Figure 15:**
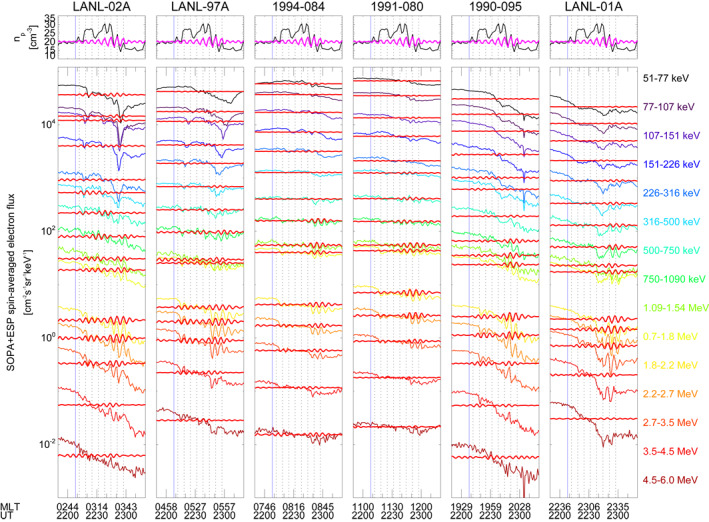
The same as Figure 14 from 21:50 UT to 23:20 UT on 9 November with data filtered in the 2.2–3.2 mHz frequency range. Vertical lines indicate the amplitude peaks for the 6.4 min periodic density structures. The blue vertical line identifies the substorm onset at 22:08 UT.

We repeated the analysis on the electron fluxes observed during the directly driven wave at ≈2.6 mHz. Figure 15 shows the measurements for the interval corresponding to the PDS II. The spectral analysis of each energy channel (not shown) revealed the global occurrence of a clear periodicity at 2.6 mHz for energies between 1.09 and 2.7 MeV. The same periodicity was identified for lower energies (51–77 and 750–1090 keV channels) in the dawn sector at the LANL–02A, LANL–97A, and 1994‐084 satellites. Closer to noon, at the 1991‐080 satellite, we identified waves at 2.9–3.1 mHz for energy channels from 51 to 1090 keV. A mixture of the two signals resulted in broad power enhancements between 2.6 and 3.1 mHz at all satellites for the 2.7–3.5 and 3.5–4.5 MeV channels and for the 500–750 keV channel at LANL–02A. Note that these periodicities agree with the two waves at 2.4–2.6 and 2.9–3.1 mHz identified in the magnetic field observations at the geostationary orbit and ground stations during the interaction with PDS II. Interestingly, Y. Liu and Zong (2015) observed similar frequency and properties in the oscillations of energetic electron fluxes at the geostationary orbit following the impact on the magnetosphere of interplanetary shock. In Figure 15 we show the filtered Wind (LANL) observations (magenta and red lines) in the 2.2–3.2 mHz (≈5.2–7.6 min) frequency range obtained with a Kaiser window filter of length 23 (37) points with stopband gain of −40 dB. In the post‐midnight region (LANL–02A) we observed a prompt response to the solar wind density fluctuations, especially at higher energies. A cross‐phase analysis between Wind density and LANL–02A electron fluxes observations showed high coherence and a phase difference of −0.86° for the 1.8–2.2 MeV channel. A progressive increase/decrease of phase difference was observed performing the same analysis down to the 750–1090 keV channel (49°) and up to the 3.5–4.5 MeV channel (−68°), respectively. The cross‐phase analysis between consecutive geostationary satellites for each energy channel between 1.09 and 2.7 MeV revealed a consistent eastward propagation of the signal resulting in anti‐phase fluxes variation at noon. Alternative visualization of the observations is available in the form of spectrograms of residual fluxes in Figure S1 of the Supporting Information.

## 7 Discussion

A train of PDSs was observed by the Wind spacecraft on 9‐10 November 2002. The larger structures occurred quasi‐periodically every ≈90 min which is a characteristic time scale of plasma release at the helmet streamer as observed in coronagraph images (Viall & Vourlidas, 2015) and predicted by recent simulations (Réville et al., 2020). According to the WSA model results, the observed solar wind parcel was at first connected to an active region and a mid‐latitude coronal hole before the crossing of a highly inclined HCS. The predicted crossing of the HCS aligns well with the observed crossing of the HCS providing confidence that our source mapping is correct. At a smaller scale, we identified clear density fluctuations at ≈2.5–2.7 mHz and broad power enhancements centered at ≈1.5 mHz and ≈1.8 mHz. These frequencies are similar to those identified in previous statistical in situ studies at 1 AU (Viall et al., 2009). The almost constant total pressure of the PDSs associated with the anticorrelation between *n*_*p*_ and B, as well as *p*_*T*_ and *p*_*B*_, is a characteristic signature of pressure balance structures (Bavassano et al., 2004; Burlaga & Ogilvie, 1970; Tu & Marsch, 1994). Signatures of conversion into compressive structures were observed at the boundary of two adjacent substructures in PDS II in which we observed an isolated increase of the total pressure. Even though some instances of PDSs have been associated with the transit of flux‐ropes (Di Matteo et al., 2019; Kepko et al., 2016), the minimum variance analysis applied to different portions of this solar wind stream did not reveal any clear signature of flux‐rope. On the other hand, the PDSs showed some rotation of the magnetic field characterized by the absence of a core field, the enhancement of the *β* value, and many of the density structures were associated with changes in *n*_*α*_/*n*_*p*_, which is set in the solar atmosphere. These properties are similar to ones observed in PDSs closer than 1 AU (Di Matteo et al., 2019) and plasmoids predicted by 3–D MHD simulation (Higginson & Lynch, 2018) suggesting that these structures are remnants of solar corona processes.

The spectral analysis of the magnetic field at the geostationary orbit and ground revealed that the interaction of the magnetosphere with solar wind periodic density structures resulted in a global modulation of the magnetosphere at the longer time scales associated with each PDS, as well as ULF waves at discrete frequencies. Kepko et al. (2020) identified PDSs in solar wind measurement from the Wind spacecraft over two solar cycles. In a conservative estimate, they determined a PDS occurrence rate of ≈60% in the slow solar wind and ≈30% in the fast solar wind. Paired with the conclusion of (Viall et al., 2009), who found one‐to‐one correspondence between periodic solar wind density fluctuations and magnetic field oscillation at the geostationary orbit for ≈50% of cases, led to the conclusion that directly driven ULF waves could occur for ≈ 30% and ≈15% of the time for slow and fast solar wind, respectively. An extensive study to provide a more accurate estimate of the driven ULF waves occurrence is underway. Table 1 summarizes our results and gives a better insight into the PDSs‐magnetosphere interaction process. A visual representation of the magnetosphere response is available in the Supporting Information as a video showing global maps of the ULF waves occurrence at selected frequency bands (similar to Figures 8, 9, 10, 11) for a 91 min running window.

**Table 1:** Ultra Low Frequency Waves Frequencies Identified at the Geostationary Orbit and Ground Observatories^a^

Variable	Second SI			PDS I			PDS II		PDSs III‐IV‐V			
Wind *n*_*p*_	(1.5)						2.6		(1.0	2.3)		
*GOES8 B*_*μ*_	**1.6**								**1.9**			
GOES8 *B*_*ϕ*_				**2.3**		4.5	***2.6***		**1.9**	*2.4*		
*GOES8 B*_*ν*_	**1.6**					3.6	***2.5*** ^b^	*3.0* ^b^ 3.4^b^		*2.4*		
*GOES10 B*_*μ*_	**1.6**			**2.4**	*2.7* ^b^		***2.7***		**1.9**			
GOES10 *B*_*ϕ*_			*4.6*				***2.7***				*3.2*	
*GOES10 B*_*ν*_							***2.7***		**1.9**		*3.2*	
*B*_*N*_ high *λ*	**(1.5)**	*3.7*	*4.6*	**2.3**	*2.6* ^b^	3.4 → 3.7^b^	***2.6*** **→ 2.4** ^b^	*3.1* → 2.9^b^				
*B*_*N*_ mid *λ*	**1.5**			**2.3**		3.4 → 3.7^b^	***2.6*** **→ 2.4** ^b^	*3.1* → 2.9	**1.8**		*3.1*	4.9
*B_N_* low *λ*	**1.5**					3.7^b^	***2.6*** **→ 2.4** ^b^	*2.9* ^b^	**1.8**			4.9
*B_E_ *high *λ*		*3.7*	*4.6*	**2.3**		3.4 → 3.7^b^	***2.6*** **→ 2.4** ^b^	*3.1* → 2.9^b^				
*B*_*E*_ mid *λ*	**(1.5)**			**2.3**		3.4 → 3.7^b^	***2.6*** **→ 2.4** ^b^	*(3.1)* → 2.9^b^	**1.8**	*2.4*		
*B_E_* low *λ*				**2.3**		3.4 → 3.7^b^	***(2.6)*** **→ 2.4** ^b^	*2.9* ^b^	**1.8**			

*Note.* →indicates a rising/decreasing tone; parenthesis indicates a lower occurrence of the waves. Values in italics and bold indicate respectively FLR and global modes.

^*a*^ For each wave mode, we reported the frequency in mHz.

^b^ frequencies for waves occurring at the border of the time interval.

The magnetospheric response to the impact of the two shocks was characterized by ULF waves with different spatial distribution and persistence. As an example, the comparison of the magnetic field *B*_*N*_ component at JAN and MAW in Figure 8a shows a similar fast damped ULF wave after the first SI (C. Wang et al., 2015), but persistent wave at different frequencies after the second SI. While the differences in the response might be related to the distinct intensity and orientation of the two shocks (Oliveira et al., 2020), strong dynamic pressure fluctuations following S2 (absent after S1) might also have triggered the waves or have provided additional energy to sustain the oscillations for a longer time. The enhanced power up to ≈2 mHz in the dynamic spectrum of the solar wind density (Figure 4) and the global occurrence of the wave at ≈1.5–1.6 mHz suggest that this mode might be directly driven by the solar wind. For the waves at higher frequencies, we identified one at ≈4.6 mHz along the toroidal component at GOES10. Di Matteo and Villante (2018) also found waves near the two higher frequencies, 3.7 and 4.6 mHz identified here.

To gain more insight into the nature of these fluctuations we used the ground observatories to investigate their polarization pattern (see Section 2 for details on the analysis), shown in Figure 12a. At the position of each station identifying a wave at a specific frequency, in either the *B*_*N*_ or *B*_*E*_ component, red/blue arrows represent right‐/left‐handed polarization, while black arrows indicate linear polarization. The polarization pattern for the wave at ≈1.5 mHz exhibited a polarization reversal across ≈12–15 MLT. We found evidence of FLR in the form of amplitude peak and a ≈180° phase variation (not shown) in the *B*_*N*_ component of the ≈3.7 mHz wave at ≈71°‐73° in the 17–19 MLT sector and ≈4.6 mHz at ≈62°‐69° in the 08–10 MLT sector, consistent with the linear polarization locations in Figure 12a (Chen & Hasegawa, 1974; Hughes & Southwood, 1976; Piersanti et al., 2012; Samson et al., 1991). The three detected waves were associated with low azimuthal wave number (Figure 13a) with values typically |*m*| < 4. These results suggest that the ≈1.5 mHz wave was directly driven by the solar wind, while the ≈3.7 mHz and ≈4.6 mHz waves were likely fast mode resonances, in which the compressional waves resulted from the interplanetary shock impact and/or the impulsive buffeting from the density structures.

At the beginning of the PDS I interval we observed waves at ≈2.3 mHz and ≈3.4 mHz. The wave at ≈2.3 mHz occurred: (a) along the compressional component at GOES10 (≈11:30 MLT) and along the *B*_*E*_ component in the dayside sector at the ground below the auroral zone and (b) along the toroidal component at GOES8 (≈15:30 MLT) and the *B*_*N*_ component along and below the auroral zone respectively in the dayside and nightside sector. This might result from the change in polarization of an Alfvénic mode as a function of MLT (Kabin et al., 2007). In fact, for observations at ground stations close to the foot‐point of the magnetic field line passing through the GOES satellites (Figure 12b), the polarization analysis revealed the change of the azimuthal wave angle from an east‐west direction to north‐south across ≈13–14 MLT. The waves occurred after the arrival of a strong IMF discontinuity, which might have generated a transient ion foreshock phenomenon that in turn could have triggered the Pc5 waves (Hartinger et al., 2014; B. Wang et al., 2020). In the second half of the PDS I interval, the increase of the frequency of the wave (see Table 1) occurred in correspondence with a *n*_*p*_ enhancement suggesting a possible role of the magnetosphere compression (Murphy et al., 2015; Takahashi & Ukhorskiy, 2007).

Examining the polarization pattern (Figure 12b) we found polarization reversal across ≈13–14 MLT for both wave modes. From the analysis of latitudinal arrays, we found evidence of FLR (not shown) for the ≈2.6 mHz wave at ≈64°–66° in the 19–21 MLT sector, consistent with the position of linear polarization in Figure 12c. For the other waves and MLT sector with linear polarization profile at high latitude, the FLR signatures were not clearly present, often with phase reversal not centered with amplitude peaks or associated with two power peaks in the ≈60°–75° latitudinal range. The azimuthal wave number for the detected waves in the first and second half of the interval (Figures 13b and 13c) showed low values, |*m*| < 4. However, note that on the night‐side the error bars reached values of |*m*| ∼ 10. Interestingly, following the impact of the interplanetary magnetic field discontinuity, there was a signature of westward and eastward propagation of the ≈2.3 mHz wave respectively before and after ≈13 MLT with *m* ∼ −2 and *m* ∼ 2, suggesting that the wave originated in this sector.

Right after the beginning of the PDS II interval, the ≈2.6 mHz wave persisted, while the ≈3.7 mHz one rapidly disappeared and was later replaced by a wave at ≈3.1 mHz. The corresponding polarization pattern in Figure 12d and the azimuthal wave numbers in Figure 13d were similar to the previous time interval with no clear signatures of FLR. Regarding the azimuthal wave number, we observed signatures of westward propagation of the ≈2.6 mHz with *m* ∼ − 3 before ≈14 MLT. At the impact of the solar wind parcel showing clear ≈2.6 mHz fluctuations, the polarization pattern of the two wave modes (Figure 12e) changed manifesting two longitudinal profiles of linear polarization in the 13–17 MLT sector respectively at *λ* ≈ 60°–66° and *λ* ≈ 73°–77°. The azimuthal wave number (Figure 13e) for the ≈2.6 mHz became closer to null values at all MLT, reflecting the global nature of the wave. The analysis of the magnetic field fluctuations along latitudinal arrays in this sector revealed two peaks in amplitude, each within the two latitude ranges, confined by ≈ 180° phase variation at both sides (not shown). The second peak at lower latitude might be related to a second resonance possibly related to a local background plasma density enhancement (Nielsen & Allan, 1983). The appearance of multiple amplitude peaks associated with polarization reversal and the mixture with FLRs is also compatible with MHD surface eigenmodes resulting from the magnetosphere compression due to the interaction with the PDSs (Nenovski, 2021; Nenovski et al., 2007).

During the PDSs III‐IV‐V interval, also characterized by substorm activity, we identified waves at four frequencies, namely ≈1.8, ≈2.4, ≈3.1, and ≈4.9 mHz. While the wave at ≈1.8 mHz showed a more global character and was related to similar fluctuations in the solar wind density, the waves at higher frequency were more localized. The narrow azimuthal extent of these waves was confirmed by observations at the geostationary orbit with the ≈2.4 mHz wave detected along *B*_*ϕ*_ and *B*_*ν*_ only at GOES8 located at ≈20 MLT and the ≈3.2 mHz wave detected along the same magnetic field components only at GOES10 located at ≈16 MLT. Note that at GOES8 the wave activity was clear with large amplitude fluctuations along the toroidal component suggesting that the satellite was moving through an FLR, and the ground observations in the same MLT sectors observed the expected amplitude peak and 180° phase variation at *λ* ≈ 67°–70° (not shown). The same analysis for the ≈3.1 mHz wave revealed FLR signatures at *λ* ≈ 60°–62° in the 14–17 MLT sector. This is also consistent with the polarization pattern in Figure 12f showing linear polarization at the same latitudes and MLT sectors. The azimuthal wave number (Figure 13f) showed values close to zero for the wave at ≈1.8 mHz reflecting its global nature. For the waves at the higher frequency we observed large *m* values in the post‐midnight sector reaching a value of *m* ∼ −10. In the dayside sector, there were no station pairs satisfying our criteria suggesting the possible high *m* values for these waves and their relation to drift or drift‐bounce resonance with injected energetic particles resulting from the substorm activity. However, note that the waves' azimuthal and latitudinal structure might be also related to the underlying magnetosphere plasma distribution rather than to the generation mechanism, as this can determine dawn/dusk asymmetry (Archer & Plaschke, 2015) and regulate the wave penetration into the inner magnetosphere (Degeling et al., 2018).

The role of the PDSs in the solar wind‐magnetosphere interaction is also related to prompt coherent modulation of energetic particles (Kepko & Viall, 2019; Tan et al., 2011). The PDSs period falls within and extends beyond the Pc5 band determining compressional ULF waves which are known to be important for energetic particle acceleration, loss, and transport, particularly in the outer radiation belts (H. Liu et al., 2016; Mann et al., 2016; Ozeke et al., 2018; Zhang et al., 2019; Zhou et al., 2015). For the event in analysis, the prompt response to the 90 min PDSs I and II at low energy in the noon region might result from the energization of the lower energy electron population. The global antiphase response of electron fluxes at higher energy instead suggests the movement of particle boundaries at lower L‐shells as the magnetosphere was compressed by solar wind PDSs. During the interaction with PDS II, the 6.4 min (≈2.6 mHz) density sub‐structures determined a prompt in phase response of electron fluxes in the post‐midnight region at LANL–02A for the 51–77 keV following the substorm onset at 22:08 UT (vertical blue line in Figure 15). Modulation of electron fluxes at energies up to tens of keV might have been a consequence of whistler‐mode chorus waves modulated by ULF waves. In the dawn sector, the ULF wave might have periodically transported at lower L shells hot electrons injected from the plasma sheet. The resulting anisotropies might have generated chorus waves at the minima of the magnetic field compressive component fluctuations (Zhang et al., 2019). The chorus waves then might have caused periodic electron precipitation at energies of ≈1–100 keV (Ma et al., 2020) resulting in subsequent decreases which would appear as electron flux variation in phase with the compression of the magnetosphere as we observed. For fluxes at higher energy, the in‐phase response for fluctuations in the 1.8–2.2 MeV channel and the increasing/decreasing phase change in the adjacent energy channels might be the result of drift resonance (Zhou et al., 2015). As a consequence, the anti‐phase fluxes variation at noon might be the result of electrons drifting eastward from the post‐midnight region. This is also suggested by the first dip in fluxes observed progressively from LANL–02A to LANL–01A. Following the approach of (Murphy et al., 2018) we estimated the azimuthal wave number from the drift resonance condition in an asymmetric dipole (Elkington et al., 1999, 2003). Considering electrons with 90° pitch angles, we used the bounce‐averaged drift period at *L* ≈ 6.6 (Komar et al., 2017; Walt, 1994), the identified frequency of the ULF wave (≈2.6 mHz), and the energy range of the affected electrons (≈1.8–2.2 MeV). We obtained *m* ≈ 2, compatible with the values in Figure 13e. In the noon and dusk regions, there was no clear increasing/decreasing phase change in the adjacent energy channels. On one hand, analysis of ground magnetometer observations in this region revealed an additional wave at 3.1 mHz. This compressional wave might present an azimuthal gradient introducing influence by the mirror effect which can also result in an anti‐phase response for electron fluxes over a broad energy range (H. Liu et al., 2016). On the other hand, we might have a different radial gradient of the phase space density profile influencing high energy electrons drift resonant interaction, especially in the aftermath of an interplanetary shock (Hartinger et al., 2020).

## 8 Summary and Conclusions

On 9‐10 November 2002, the Wind spacecraft observed PDSs with periodicities ranging from several minutes to ≈90 min. These PDSs impacted the magnetosphere resulting in a number of different dynamics in the magnetosphere, including the direct driving in the ULF waves, FLRs, and local changes in radiation belt particle flux. The pressure balance nature of these structures together with the corresponding enhancements of the *β* value and *n*_*α*_/*n*_*p*_ suggest they were formed through solar corona processes, consistent with previous work (Di Matteo et al., 2019; Kepko et al., 2016; Viall & Vourlidas, 2015). Using the WSA model, we identified the source of this solar wind stream as an active region and a mid‐latitude coronal hole close to a highly inclined HCS. This is the first time that the solar source region of PDSs has been robustly identified for an event in which they drove magnetospheric dynamics.

The magnetospheric response to the PDSs in terms of ULF waves revealed a combined occurrence of directly driven and triggered wave modes:

1. The longer fluctuations, corresponding to frequencies lower than ≈ 1 mHz, resulted from a forced breathing process. The resultant magnetic field variations at geostationary orbit, simulated as a series of equilibrium states of the magnetosphere with the T04 model, reproduced the fluctuations in the dayside sector well
2. At higher frequencies, we observed globally with ground magnetometers four wave modes: the ≈1.5 mHz after the second SI; the ≈2.3 mHz during the PDS I; the ≈2.6 mHz during the PDS II; and the ≈1.8 mHz during the PDSs III‐IV‐V. The fluctuations at ≈2.6 mHz was the only one clearly identified in the dynamic spectrum of the solar wind density and indeed it manifested in the magnetic field at the geostationary orbit and everywhere at the ground, consistent with a forced breathing mode. The ≈1.5 mHz and ≈1.8 mHz were also related to the solar wind density, whose dynamic spectrum showed strong enhancements at similar frequencies. The ≈2.3 mHz wave showed signs of propagation away from 13 to 14 MLT and followed the arrival of an interplanetary magnetic field discontinuity, which marked the boundary of the first PDS, suggesting the role of ion foreshock phenomena in the triggering of this wave (Hartinger et al., 2014; C.‐P.Wang et al., 2017; B. Wang et al., 2020)
3. The other waves at a higher frequency, ≳2 mHz, were mostly localized to mid and high latitude ground observatories in the post‐noon MLT sector, in some cases confirmed with observations at the geostationary orbit and associated with FLR. The occurrence at high latitude from afternoon to postmidnight is consistent with a recent analysis of Pc5 wave in observations from Super Dual Auroral Radar Network (Norouzi‐Sedeh et al., 2015; Shi et al., 2018). Waves showing right‐/left‐handed polarization before/after the ≈13–14 MLT sector are consistent with anti‐sunward propagating disturbances whose origin lies in the solar wind (Hughes, 1994). This also manifested in the corresponding low azimuthal wave number, which was either close to zero or exhibited slightly negative/positive values before/after 13–14 MLT. The ULF waves in the afternoon sector showed fewer signatures of FLRs, but when identified they might result from the impulsive buffeting from the solar wind and/or waveguide mode weakly coupled with FLR (Chisham & Orr, 1997; Fenrich et al., 1995; Mann & Wright, 1999; Rostoker & Sullivan, 1987; Ziesolleck & McDiarmid, 1995) or drif/drift–bounce resonance process (Claudepierre et al., 2013; Glassmeier et al., 1999; James et al., 2013; Yeoman et al., 2010). Note that the wave's azimuthal and latitudinal structure might be also related to the underlying magnetosphere plasma distribution (Archer & Plaschke, 2015; Degeling et al., 2018)

In this case study, we have also shown that while dynamic pressure variations at long time scales (≲1 mHz) directly drove ULF waves at similar frequencies, they influenced the properties of waves at a higher frequency, but not their occurrence (Hartinger et al., 2014). Therefore, we might have intervals with simultaneous global and localized ULF waves which can be important in determining the energy exchange with radiation belt electrons in an extended energy range (Hao et al., 2020). Observations of the electron particle fluxes at the geostationary orbit from six LANL satellites, covering different LT sectors and a wide energy range, manifested prompt modulations from the 90 min PDSs as a possible result of local energization at low energies in the noon sector and movement of particle boundaries at high energies. The electron flux modulation resulting from the solar wind driven 2.6 mHz ULF wave show possible signatures of Chorus (whistler mode) waves modulation in the post‐midnight region at low energies and drift resonance at high energies.

The structure of ULF waves in the Pc5 frequency range play a fundamental role in the dynamic of radiation belts (Mann et al., 2016; Ozeke et al., 2018; Zong et al., 2017), supplying relativistic electrons due to radial diffusion, adiabatic acceleration, drift and drift‐bounce resonance acceleration (Baker et al., 2018; Degeling et al., 2008; Elkington et al., 2003, 1999; Elkington & Sarris, 2016; Mathie & Mann, 2001; Ozeke & Mann, 2008; Regi et al., 2015; Schulz & Lanzerotti, 1974; Yeoman & Wright, 2001; Zong et al., 2017). Especially in the resonant interaction, the distinction between the discrete and broad‐band nature of the waves is fundamental (Murphy et al., 2020). Previous studies on this subject were limited by the spectral analysis procedures that often were restricted to the selection of the most relevant peak in the power spectrum, possibly within a set of discrete ULF waves. This becomes even more critical if the spectral analysis procedure is unable to resolve broad power spectrum enhancements due to discrete waves at close frequencies (Di Matteo & Villante, 2017). In this regard, with this case study, we showed that our new methodology constitutes a promising tool for a detailed investigation of the discrete ULF waves properties and preferential location.

## Supporting information

Supporting Information S1

Table S1

Movie S1

## Data Availability

Data used in this study are available from the SuperMAG web site (https://supermag.jhuapl.edu/), see Supporting Information for full acknowledgment. The SOPA and EPS data used in this study are available at https://zenodo.org/record/5758163 (Reeves, 2021). Part of data access and processing was done using SPEDAS V4.1 (Angelopoulos et al., 2019).
