# Supplementary material for: On Differentiating Multiple Types of ULF Magnetospheric Waves in Response to Solar Wind Periodic Density Structures
Source: J Geophys Res Space Phys. 2022 Mar 22;127(3):e2021JA030144. doi: 10.1029/2021JA030144 (PMC9285707; doi:10.1029/2021JA030144)
Supplement: Supplementary file 1 — Supporting Information S1 [file JGRA-127-0-s001.pdf]

Supporting Information for "On differentiating multiple types of ULF magnetospheric waves in response to solar wind periodic density structures"

S. Di Matteo[1,2], U. Villante[3,4], N. Viall[2], L. Kepko[2], S. Wallace[2]

[1]Physics Department, The Catholic University of America, Washington, DC 20664, USA.

[2]NASA - Goddard Space Flight Center, Greenbelt, MD 20771, USA.

[3]Department of Physical and Chemical Sciences, University of L'Aquila, L'Aquila, Italy.

[4]Consorzio Area di Ricerca in Astrogeofisica, L'Aquila, Italy.

Contents of this file

1. Figure S1

Additional Supporting Information (Files uploaded separately)

1. Caption for large Table S1
2. Caption for Movie S1

Corresponding author: S. Di Matteo, NASA – Goddard Space Flight Center, Solar Physics Laboratory, Code 671, Building 21, Greenbelt, MD 20771, USA. (simone.dimatteo@nasa.gov)

Introduction

The magnetic field response at ground was investigated using 181 ground observatories from the SuperMAG collaboration (Gjerloev, 2012). Information about the stations used in our analysis are in Table S1. We apply our spectral analysis procedure (Di Matteo et al., 2020) to the the north-south (B_N) and east-west (B_E) magnetic field component at each observatory to reveal the occurrence of ULF waves at discrete frequencies. The movie S1 shows an overview of the results from 17:09 UT on November 9, 2002, to 01:30 UT on November 10, 2002. The analysis is performed for a running 91-minute interval with 3-minute steps. The parameters for the spectral analysis are the ones described in the main text. The maps are qualitative representation of the global power distribution obtained interpolating on a regular grid (Isaaks & Srivastava, 1989) the integrated power spectrum over five frequency ranges: (I) ≈1.3–2.1 mHz; (II) ≈2.2–2.8 mHz; (III) ≈2.9–3.3 mHz; (IV) ≈3.4–3.9 mHz; (V) ≈4.4–5.1 mHz. We indicate the occurrence of a discrete ULF waves with white/black dots at the location of the ground observatory. Note that in dark blue regions of the maps (i.e., very low values of integrated power), short isolated identifications are more likely to results from the selection of false positives.

Solar wind periodic density structures at ≈2.6 mHz directly drove global magnetospheric field fluctuations at similar frequency between 22:00 and 23:30 UT. During the same interval we observed signatures of drift resonance in energetic electron fluxes obtained from geostationary satellites. Figure S1 shows results similar to Figure 15 in the main text but in form of spectrograms of the residual fluxes (Claudepierre et al., 2013) in which the background flux at each energy channel has been evaluated with a 12 min running average.

Extended list of acknowledgments. For the ground magnetometer data we gratefully acknowledge: INTERMAGNET, Alan Thomson; CARISMA, PI Ian Mann; CANMOS, Geomagnetism Unit of the Geological Survey of Canada; The S-RAMP Database, PI K. Yumoto and Dr. K. Shiokawa; The SPIDR database; AARI, PI Oleg Troshichev; The MACCS program, PI M. Engebretson; GIMA; MEASURE, UCLA IGPP and Florida Institute of Technology; SAMBA, PI Eftyhia Zesta; 210 Chain, PI K. Yumoto; SAMNET, PI Farideh Honary; IMAGE, PI Liisa Juusola; Finnish Meteorological Institute, PI Liisa Juusola; Sodankylä Geophysical Observatory, PI Tero Raita; UiT the Arctic University of Norway, Tromsø Geophysical Observatory, PI Magnar G. Johnsen; GFZ German Research Centre For Geosciences, PI Jürgen Matzka; Institute of Geophysics, Polish Academy of Sciences, PI Anne Neska and Jan Reda; Polar Geophysical Institute, PI Alexander Yahnin and Yarolav Sakharov; Geological Survey of Sweden, PI Gerhard Schwarz; Swedish Institute of Space Physics, PI Masatoshi Yamauchi; AUTUMN, PI Martin Connors; DTU Space, Thom Edwards and PI Anna Willer; South Pole and McMurdo Magnetometer, PI's Louis J. Lanzarotti and Alan T. Weatherwax; ICESTAR; RAPIDMAG; British Artarctic Survey; McMac, PI Dr. Peter Chi; BGS, PI Dr. Susan Macmillan; Pushkov Institute of Terrestrial Magnetism, Ionosphere and Radio Wave Propagation (IZMIRAN); MFGI, PI B. Heilig; Institute of Geophysics, Polish Academy of Sciences, PI Anne Neska and Jan Reda; University of L'Aquila, PI M. Vellante; BCMT, V. Lesur and A. Chambodut; Data obtained in cooperation with Geoscience Australia, PI Andrew Lewis; AALPIP, co-PIs Bob Clauer and Michael Hartinger; MagStar, PI Jennifer Gannon; SuperMAG, PI Jesper W. Gjerloev; Data obtained in cooperation with the Australian Bureau of Meteorology, PI Richard Marshall.

Table S1. List of geomagnetic observatories. From the left: IAGA code, station name, chain name, geographic latitude and longitude, magnetic latitude and longitude.

Movie S1. Top left: sym-H and AE indices compared with the solar wind dynamic pressure shifted forward of 27 minute. The vertical lines are the same of Figure 7. The green patch indicates the running 91–minute time interval over which we apply our spectral analysis. Panel I–V, global maps of the integrated power spectrum for the B_N (left) and B_E (right) components in five frequency ranges, namely: (I) ≈1.3–2.1 mHz; (II) ≈2.2–2.8 mHz; (III) ≈2.9–3.3 mHz; (IV) ≈3.4–3.9 mHz; (V) ≈4.4–5.1 mHz. At the locations of the ground observatories used for the analysis (grey dots), white and black dots indicate the identification of a wave with the γ and γ+F test, respectively, within 10 minute from the map time. The dashed lines represent the auroral oval boundaries.

References

Claudepierre, S. G., Mann, I. R., Takahashi, K., Fennell, J. F., Hudson, M. K., Blake, J. B., ... Wygant, J. R. (2013). Van Allen Probes observation of localized drift resonance between poloidal mode ultra-low frequency waves and 60 kev electrons. *Geophysical Research Letters*, *40*(17), 4491-4497. doi: 10.1002/grl.50901

Di Matteo, S., Viall, N. M., & Kepko, L. (2020). *SPD_MTM: a spectral analysis tool for the SPEDAS framework.* Zenodo. Retrieved from `https://zenodo.org/record/3703168` doi: 10.5281/zenodo.3703168

Gjerloev, J. W. (2012). The SuperMAG data processing technique. *Journal of Geophysical Research: Space Physics*, *117*(A9). doi: 10.1029/2012JA017683

Isaaks, E. H., & Srivastava, R. M. (1989). *An introduction to applied geostatistics.* New York: Oxford University Press. doi: 1969drea.book.....B

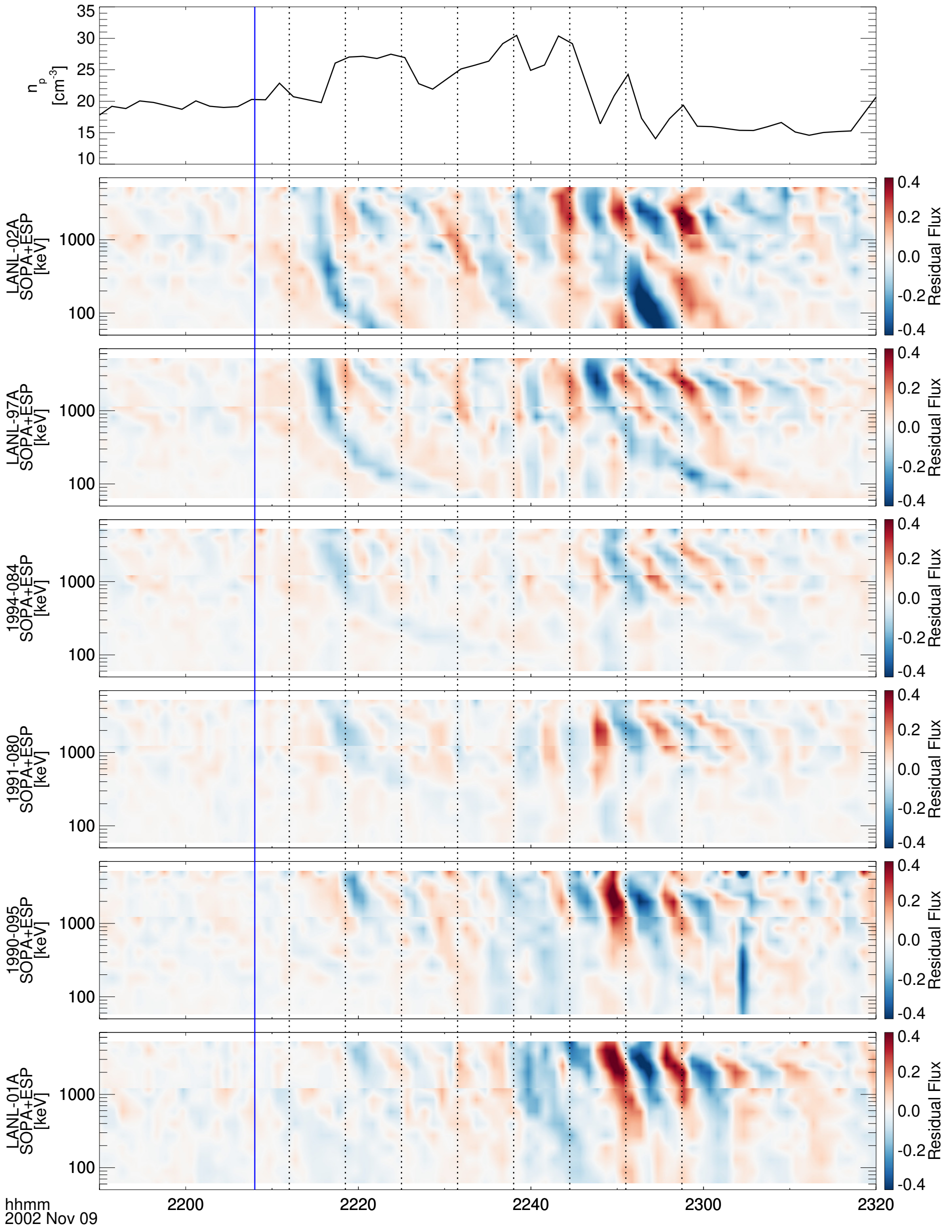

Figure S1. One-minute spectrogram of electron particle residual flux at the geostationary orbit based on 15 differential energy channels from six LANL satellites compared with the solar wind proton density (top panel) for the time interval from 21:50 UT to 23:20 UT on November 9, 2002. The vertical lines identify peaks for the 6.4 min min solar wind periodic density structures. The blue vertical line identifies the substorm onset at 22:08 UT.
